# Majorana edge reconstruction and the *ν* = 5/2 non-Abelian thermal Hall puzzle

**DOI:** 10.1073/pnas.2608514123

**Published:** 2026-11-05

**Authors:** Tevž Lotrič, Taige Wang, Michael P. Zaletel, Steven H. Simon, S. A. Parameswaran

**Affiliations:** ^a^Rudolf Peierls Centre for Theoretical Physics, Oxford OX1 3PU, United Kingdom; ^b^ Department of Physics, University of California at Berkeley, Berkeley, CA 94720; ^c^ Material Science Division, Lawrence Berkeley National Laboratory, Berkeley, CA 94720; ^d^ Department of Physics, Harvard University, Cambridge, MA 02138; ^e^ Materials Research Laboratory, Massachusetts Institute of Technology, Cambridge, MA 02139

**Keywords:** fractional quantum Hall effect, Majorana fermions, topological quantum physics

## Abstract

Pioneering thermal transport measurements on two-dimensional electron gases in high magnetic fields have demonstrated that the quantized Hall state at filling factor *ν* = 5/2 has a thermal Hall conductance *κ* quantized in half-integer multiples of $\kappa_{0}=\pi^{2}k_{B}^{2}T/3h$. Half-integer $\kappa /\kappa_{0}$ is a signature of neutral Majorana edge modes, in turn linked to the presence of non-Abelian anyon excitations in the bulk. However, the experimentally observed value of *κ* corresponds to the “PH-Pfaffian” state, in tension with numerical studies which instead favor either the Pfaffian or the AntiPfaffian. A variety of mechanisms have been invoked to explain this discrepancy, but have been either ruled out by further experiments or else involve fine-tuning. Building on density-matrix-renormalization group studies of physically realistic edges and analytic calculations of edge structure, we propose an alternative resolution of this puzzle involving an “edge reconstruction” solely involving the neutral Majorana sector of the theory. Such a Majorana edge reconstruction can “screen” a Pfaffian or AntiPfaffian bulk, so that transport signatures become indistinguishable from those of the PH-Pfaffian. We argue that this physically natural scenario is consistent with experiment.

## Significance

Many-body quantum systems can exhibit special collective behaviors offering routes toward robust quantum technologies. A well-studied example is a two-dimensional electronic state formed in strong magnetic fields, whose fractionalized excitations are expected to leave clear signatures in how heat flows along the system’s boundaries. For years, experiments measuring this heat flow have contradicted theoretical predictions despite extensive numerical and analytical work. We show that this conflict can be resolved by previously unrecognized boundary mechanics involving emergent neutral modes. We identify “Majorana edge reconstruction” as a mechanism by which boundary behavior can obscure the nature of the underlying quantum state. This resolves a long-standing experimental puzzle and highlights the role of boundaries in determining observable properties of topological quantum systems.

The nature of the fractional quantized Hall (FQH) plateau at Landau level (LL) filling factor *ν* = 5/2 has been a matter of intense debate since its discovery (1). Several distinct incompressible phases of matter have been proposed to explain the experiments, with the preponderance of numerical evidence (2–18) indicating that the most likely candidates are the Moore-Read Pfaffian (Pf) (19) or its particle–hole conjugate, the AntiPfaffian (APf) (20, 21) phase of matter. However, a groundbreaking series of experiments (22–25) have instead consistently favored an interpretation in terms of a third phase, the PH-Pfaffian (PHPf) (20, 26). All three possibilities host quasiparticle excitations with non-Abelian statistics, meaning that they are candidates for topological information processing (27), and hence are particularly exciting for fundamental and practical reasons.

In light of this, the tension between theory and experiment is unsatisfactory. Although it is possible that the theories fail to capture some essential feature favoring PHPf in experiments, the situation is in stark contrast to the remarkable agreement at several other filling factors whose quasiparticles have Abelian fractional statistics (28). An early attempted reconciliation, advanced by one of us, relied on the observation that experiments only probe physics at the edge, and suggested that incomplete equilibration of a Majorana edge mode would explain the measured thermal conductance (29–32). Although initially promising, this explanation—which only applies to the APf—has been significantly challenged, particularly given further experiments (23–25). Further versions of the nonequilibrated edge story have been proposed in ref. 33, but that explanation still relies on some restrictive assumptions if one demands an APf bulk.

An alternative approach focused instead on the bulk, and explored a scenario wherein the effect of interactions on disorder-induced puddles of Pf and APf (34–36) could lead to PHPf physics at long wavelengths. However, the necessary conditions seem quite fine-tuned (34), making it less plausible as a realistic explanation of experiments. One further possibility suggests a compressible ground state in the bulk, which can in some cases still display a quantized thermal Hall response at finite temperature via a mechanism proposed in ref. 37. But exact details of why a compressible state would be energetically favorable at ν=5/2 in light of the numerical evidence in favor of the gapped Pf/APf bulk states remain unclear.

**Fig. 1.**
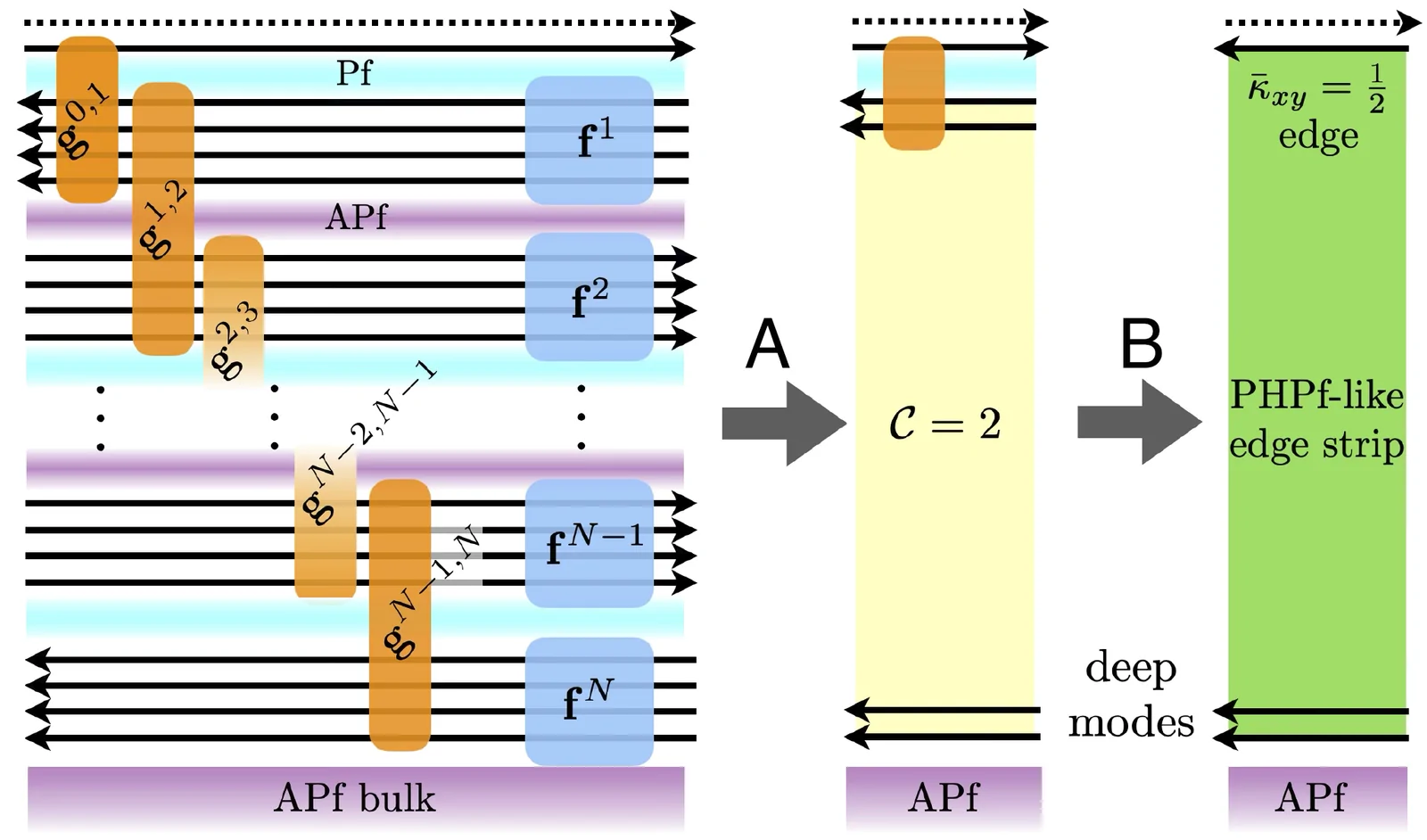
Majorana edge reconstruction. Majorana mode quadruplets (solid arrows) bound to Pf/APf domain walls near the edge (*A*) first gap out “pairwise” to produce a C=2 strip, whose edge modes in turn (*B*) couple with the charge (dashed) and Majorana (solid) mode at the physical boundary to produce an effective ${\overset{¯}{\kappa }}_{\mathrm{xy}}=1/2$ edge and a pair of “deep modes” separated by a mesoscopic strip of PHPf. Edge transport measurements are insensitive to the deep modes that do not equilibrate with physical edge modes due to their large separation from them.

Here we propose a natural resolution to the conflict between theoretical predictions and experimental observations at ν=5/2. Our approach builds on the numerical observation, via density-matrix renormalization group (DMRG) studies, of a form of reconstruction at the edge of a half-filled LL. For experimentally realistic confining potentials, the incompressible QH liquid exhibits very strong density oscillations extending over $L_{e}∼40\ell_{B}$ into the bulk (recall that the magnetic length $\ell_{B}={\left(\hbar /eB\right)}^{1/2}$ is the natural scale for a LL in magnetic field *B*). Surprisingly, the chirality of the entanglement spectrum oscillates in tandem with the electron density at the location of the entanglement cut. This finding hints at the formation of alternating stripes of Pf and APf near the edge. A similar structure for the entire bulk sample was proposed in ref. 38, but in the present scenario the stripe pattern is not a property of a stable bulk phase but only exists in a finite region near the edge. Each of the series of Pf/APf interfaces suggested by this reconstruction hosts four Majorana modes, with successive quadruplets propagating in alternating directions. Over a wide range of physically reasonable parameters, we show analytically, within an effective model, that backscattering between adjacent quadruplets and between the outermost quadruplet and the original edge modes results in an effective PH-Pfaffian edge. We find that this PHPf-like edge is nevertheless consistent with either a bulk Pf or bulk APf phase, due to the emergence of a pair of chiral modes deep in the bulk that combine with it to satisfy the mandates of the bulk-boundary correspondence, as illustrated for the edge structure of an APf bulk in Fig. 1. We show that edge thermal measurements are insensitive to the deep modes: the emergent mesoscopic length scale $L_{e}$ “screens” the bulk Pf or APf, so that edge experiments diagnose PHPf behavior. Remarkably, this picture, although blending aspects of both previous proposed resolutions, in contrast to them does not require fine-tuning and—via the emergent mesoscopic scale $L_{e}$—gives a concrete physical mechanism for the lack of equilibration between the spatially separated edge modes.

Ref. 33 previously proposed a PH-Pfaffian edge separated from a Pfaffian or anti-Pfaffian bulk, while ref. 34 considered a sufficiently thick Pf/APf domain wall near the boundary that could similarly produce the observed thermal Hall response. Neither work identified a microscopic mechanism for generating such an intervening structure. Here, we provide a concrete physical mechanism behind the emergence of a mesoscopic scale $L_{e}$ and the formation of a PHPf strip.

In the remainder, we review the physics of the ν=5/2 edge in Section 1, and then present our numerical results that motivate the edge reconstruction scenario in Section 2. We then provide an analytically tractable theory for the reconstruction in Section 3. We then use this to rationalize various features of the numerical data in Section 4, before providing an analysis of the thermal conductance measurements in light of the reconstruction in Section 5. We close in Section 6 by discussing the implications of our results for future interferometric probes of non-Abelian statistics.

## 1. The *ν* = 5/2 Edge

We begin by summarizing the different possible ν=5/2 edge structures, focusing on the Pfaffian-like states. While other states, including the Halperin-331 are in principle possible at ν=5/2, the half-integer thermal Hall conductance is most naturally explained by the Pfaffian family (39). A more detailed overview is given in *SI Appendix*.

Throughout, we will ignore the two filled LLs, and focus on the half-filled level, which contributes $\sigma_{\mathrm{xy}}={\overset{¯}{\sigma }}_{\mathrm{xy}}\sigma_{0}$ and $\kappa_{\mathrm{xy}}={\overset{¯}{\kappa }}_{\mathrm{xy}}\kappa_{0}$ to the Hall and thermal Hall conductance. (This is equivalent to the physical edge between the half-filled level and the *ν* = 2 integer QH state.) Here we have introduced the natural units $\sigma_{0}=e^{2}/h$ and $\kappa_{0}=\pi^{2}k_{B}T/3h$ for the Hall and thermal Hall conductances, so that we can work with the dimensionless quantities ${\overset{¯}{\sigma }}_{\mathrm{xy}}$ and ${\overset{¯}{\kappa }}_{\mathrm{xy}}$ henceforth. The Hall conductance is fixed to ${\overset{¯}{\sigma }}_{\mathrm{xy}}=1/2$; in the edge-state picture, this corresponds to a single chiral charge-$\frac{1}{2}$ boson propagating “downstream,” whose Lagrangian

[1] $$\begin{array}{c} L_{c}\left[\phi \right]=-\frac{2}{4\pi }\left(\partial_{x}\phi \right)\left(\partial_{t}\phi +v_{\phi }\partial_{x}\phi \right)+\ldots \end{array}$$

is of the Luttinger liquid form, where “…” denotes higher-order terms that are irrelevant in the renormalization group (RG) sense. In contrast, there are different possibilities for ${\overset{¯}{\kappa }}_{\mathrm{xy}}$; since the charge mode gives a fixed downstream contribution ${\overset{¯}{\kappa }}_{\mathrm{xy}}^{c}=1$, edges with different ${\overset{¯}{\kappa }}_{\mathrm{xy}}$ are distinguished by the presence of |n| chiral neutral Majorana modes that may propagate either all downstream (parallel to the charge mode, *n* > 0) or all upstream (antiparallel to it, *n* < 0). These may be described by

[2] $$\begin{array}{c} L_{\text{M}}^{n}\left[\left\{\psi_{l}\right\}\right]=\sum_{l,l^{\prime }=1}^{\left|n\right|}\psi_{l}\left(i\partial_{t}\delta_{ll^{\prime }}+h_{ll^{\prime }}^{\text{sign}(n)}\left(v,f\right)\right)\psi_{l^{\prime }}+\ldots , \end{array}$$

where $h_{ll^{\prime }}^{\pm 1}\left(v,f\right)=i\left[\pm v_{ll^{\prime }}\partial_{x}+f_{ll^{\prime }}\right]$, $v_{ll^{\prime }}=v_{l}\delta_{ll^{\prime }}\geq 0$ is a diagonal matrix of mode speeds, $f_{ll^{\prime }}=-f_{l^{\prime }l}$ is an antisymmetric matrix of intermode couplings, and as before ‘…’ denotes less RG-relevant terms (at least quartic in Majoranas or quadratic in derivatives). Since all |n| modes are chiral, f cannot gap them and therefore $L_{\text{M}}^{n}$ contributes ${\overset{¯}{\sigma }}_{\mathrm{xy}}=0$ and (assuming full equilibration) ${\overset{¯}{\kappa }}_{\mathrm{xy}}=n/2$.

The simplest case is an edge with a single “downstream” Majorana mode, with Lagrangian $L_{\text{Pf}}=L_{c}+L_{\text{M}}^{1}$ and hence ${\overset{¯}{\kappa }}_{\mathrm{xy}}={\overset{¯}{\kappa }}_{\mathrm{xy}}^{c}+\frac{1}{2}=\frac{3}{2}$. This edge theory corresponds to a bulk in the non-Abelian QH phase described by the Moore-Read “Pfaffian” wavefunction (19). We remark that this describes only the minimal construction for this edge, and other descriptions of the Pfaffian edge may work better for certain scenarios, as the edge may undergo a phase transition (40).

To motivate the other possible edge structures, we observe that under a particle–hole transformation P within a LL, we have $\left({\overset{¯}{\sigma }}_{\mathrm{xy}},{\overset{¯}{\kappa }}_{\mathrm{xy}}\right)\overset{P}{\to }\left(1-{\overset{¯}{\sigma }}_{\mathrm{xy}},1-{\overset{¯}{\kappa }}_{\mathrm{xy}}\right)$. For the Pfaffian, $\left({\overset{¯}{\sigma }}_{\mathrm{xy}},{\overset{¯}{\kappa }}_{\mathrm{xy}}\right)=\left(1/2,3/2\right)\overset{P}{\to }\left(1/2,-1/2\right)$. Since the transformed value of ${\overset{¯}{\sigma }}_{\mathrm{xy}}$ remains consistent with a downstream charge mode with ${\overset{¯}{\kappa }}_{\mathrm{xy}}^{c}=1$, we conclude that the particle–hole transformed edge consists of a single charge mode and three upstream neutral Majorana modes. Thus, after a particle–hole transformation, the edge is described by the Lagrangian $L_{\text{APf}}=L_{c}+L_{\text{M}}^{-3}$; evidently, this is distinct from $L_{\text{Pf}}$ and corresponds to a distinct bulk phase of matter, captured by the so-called “AntiPfaffian” state (20, 21) (which may be obtained from the Pfaffian by applying P in the bulk).

The final possibility is an edge invariant under P, which requires that $\left({\overset{¯}{\sigma }}_{\mathrm{xy}},{\overset{¯}{\kappa }}_{\mathrm{xy}}\right)=\left(1/2,1/2\right)$. It is straightforward to see that such an edge must consist of a downstream charge mode and a single upstream Majorana mode, $L_{\text{PH}}=L_{c}+L_{\text{M}}^{-1}$, which corresponds to the “PH-symmetric Pfaffian” phase introduced in ref. 26.

Armed with this background, we now describe the central issue to be addressed. The thermal transport experiments (22, 25) as well as edge noise measurements (24) are consistent with PHPf. In contrast, numerical studies of the bulk phase—which reliably match experiments at other filling factors—consistently support Pf or APf, but never PHPf (2–16). As noted above, proposed resolutions involving poorly equilibrated edges or bulk disorder are unsatisfactory, so for guidance we turn to a numerical study of the structure of a physically realistic edge.

The ν=5/2 problem is in many ways a richer and more challenging one than many filling fractions, as the standard composite fermion picture does not immediately suggest a clear ground state. As such, the good agreement between theory and experiment at other fractions does not necessarily need to translate to ν=5/2. To truly solve the half-filled problem, one has to account for Landau level mixing and possibly disorder—because of this increased complexity, a breakdown of all known numerical methods cannot strictly be ruled out, even if it is unlikely. In the present work, we take the position that the numerical calculations favoring either Pf or APf order in the bulk do capture the relevant energetic competition correctly. We also note that electronic experimental probes have been attempted (41–44). These results are consistent with non-Abelian order, but the outcomes do not distinguish between different non-Abelian orders as sharply as the thermal Hall transport measurements.

Another indication of the ν=5/2 topological order can be found in the Levin–Halperin sequence of “daughter states” emerging when tuning *ν* to nearby fractions (45). The observed plateaus at ν=2+6/13 (46) and independently at ν=2+7/13 and ν=2+8/17 (47) can be interpreted as daughters of APf and Pf parents, respectively, but never as arising from a PH-Pf parent state (48). These sequences do not strictly rule out a PH-Pf state at ν=5/2, but are most naturally accommodated in a construction with a Pf/APf bulk, such as the present one.

## 2. Methods: Edge Reconstruction via DMRG

Edge reconstruction is a ubiquitous response of quantum Hall edges to soft confinement. Whenever the external potential varies smoothly on the scale of the magnetic length $\ell_{B}$, the system can lower its Coulomb energy by breaking the edge into several narrow incompressible strips (49–55). This “composite edge” supports a larger set of gapless modes than would be inferred from the bulk topological order alone; however, the total electrical Hall conductance ${\overset{¯}{\sigma }}_{\mathrm{xy}}$ and the thermal Hall conductance ${\overset{¯}{\kappa }}_{\mathrm{xy}}$ remain pinned as long as all the modes from all subedges have fully equilibrated (56). The equilibration scale is set by the coupling between subedges, in turn controlled by microscopic details such as the strength of interactions and disorder and the spatial separation of the charged and neutral modes, which is typically a few magnetic lengths at most.

At filling factor ν=5/2 the reconstruction becomes more intricate. Because the half-filled second Landau level supports several nearly degenerate incompressible phases, one can imagine a scenario in which different phases are locally realized as one moves inward from the sample edge. Boundaries between these phases generate subtle electrostatic effects. For instance, a Pfaffian–Anti-Pfaffian domain wall can carry a finite dipole moment, which Coulomb interactions and the external confining potential can selectively stabilize, thereby reshaping the edge profile (57, 58).

To evaluate the possibility of such reconstructions, we study an edge between the ν=5/2 and *ν* = 2 states under realistic electrostatic conditions using the “defect-DMRG” method of ref. 59. An infinitely long cylinder with circumference $L_{x}$ is partitioned such that far to the left of a wide central region the state relaxes to the *ν* = 2 integer quantum Hall vacuum, whereas far to the right it relaxes to the ν=5/2 APf phase, realizing the edge of interest in the large central region.

**Fig. 2.**
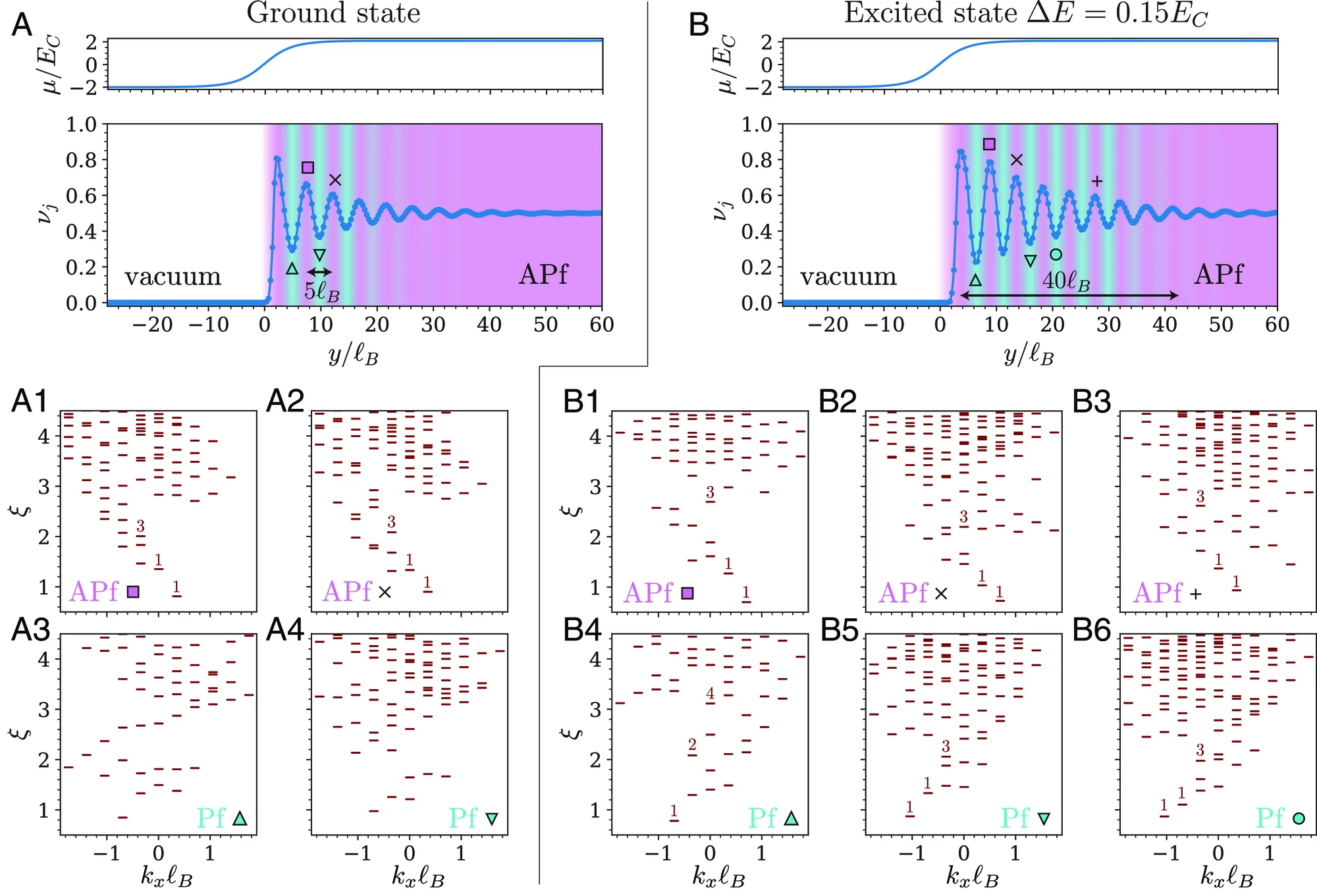
Edge reconstruction in DMRG. (*A*) Ground state with background chemical potential as in *Top* panel. (*B*) A low-lying excited state in the momentum sector $\delta k_{x}=-15.5\times 2\pi /L_{x}$ relative to the ground state. *Top* panels: orbital occupation $\nu_{j}$ and local chemical potential μ(y)=−U(y). The confining potential induces charge-density oscillations that penetrate roughly $L_{e}\approx 40\ell_{B}$ into the bulk with spatial period $5\ell_{B}$. Regions are shaded blue (Pf) or red (APf) according to the chirality inferred from the orbital entanglement spectrum (OES). *Bottom* panels: OES at representative cuts. Antichiral level counting (1,1,3,⋯) or (1,2,4,⋯) appears in (*A1* and *A2*) and (*B1*–*B3*), matching the APf edge theory. Chiral counting in (*B4*–*B6*) follows the same counting, consistent with Pf; the ground-state segments (*A3* and *A4*) are likewise chiral, though their level counting remains inconclusive. Results were obtained on a cylinder of circumference $L_{x}=18\ell_{B}$ with gate distance $d=5\ell_{B}$, chemical potentials $\mu_{L}=-2E_{C}$ and $\mu_{R}$ at the middle of the bulk gap, and bond dimension χ=2,048.

The precise model is as follows. To facilitate convergence, long-range Coulomb interactions are screened by metallic gates at z=±d, giving $V_{d}\left(q\right)=\;\tanh\left(qd\right)2\pi /q$, in units of $E_{C}=e^{2}/\left(4\pi \varepsilon \ell_{B}\right)$ units. The bottom gate is kept grounded, while the top gate is split into a left and right at voltages $V_{L}$ and $V_{R}$. Solving the Laplace equation for this split-gate geometry produces a smoothed step-like potential μ(y)=eϕ(y) at the 2DEG (Fig. 2 and *SI Appendix*), resulting in Hamiltonian

[3] $$\begin{array}{c} H=\frac{1}{2}\int drdr^{\prime }\;:\rho \left(r\right)V_{d}\left(r-r^{\prime }\right)\rho \left(r^{\prime }\right)\;:-\int dr\mu \left(r\right)\rho \left(r\right), \end{array}$$

where *ρ* is the electron density and : : denotes normal ordering. The interactions and potential are projected into the *N* = 1 LL, so henceforth the filling *ν* is measured relative to the inert *ν* = 2 background.

On the APf side, $V_{R}$ is adjusted to pin the chemical potential to the middle of APf gap, $\mu_{R}=\frac{4}{2}(\Delta_{+e/4}-\Delta_{-e/4})$, where $\Delta_{\pm e/4}$ are the energies to add or remove an e/4 quasiparticle, which we independently measured using excited-state DMRG. On the vacuum side we merely require the chemical potential to lie within the (large) *ν* = 2 cyclotron gap $-\hbar \omega_{c}<\mu_{L}<0$ (here *μ* is measured relative to ν=2+ϵ). This leaves a range of admissible $V_{L}$, which can be used to tune the sharpness of the edge. Detailed scans confirm that the qualitative reconstruction is insensitive to the precise choice of $V_{L}$ *SI Appendix*.

To obtain the ground state in the presence of the edge using DMRG, we first obtain the bulk ground states at *ν* = 0 and ν=1/2 filling of an *N* = 1 LL; the latter case produces the APf. We then consider a matrix product state (MPS) ansatz in which the MPS tensors far to the left are taken from the *ν* = 0 ground state, and far to the right are taken from the ν=1/2 APf ground state, while $N_{s}$ tensors in an intermediate window between them are variationally optimized to approximate the ground state (59). To speed convergence, we initialize the intermediate window with a block of vacuum tensors followed by APf tensors. Changing the location at which the initial ansatz switches from APf to vacuum changes the total charge; subsequent DMRG sweeps preserve this charge, allowing us to sweep through the E(Q) landscape.

Fig. 2*A* shows the ground-state charge density profile with $L_{x}=18\ell_{B}$, gate spacing $d=5\ell_{B}$, chemical potential $\mu_{L}=-2E_{C}$, and bond dimension χ=2,048. Pronounced oscillations of period $\lambda ∼5\ell_{B}$ persist over a remarkably long decay length $L_{e}∼40\ell_{B}$. To reveal the local topological structure underlying the density oscillation, we examine the orbital entanglement spectrum (OES) (60) for cuts at different locations.

Deep in the APf bulk the orbital entanglement spectrum (OES) displays the expected antichiral counting of the APf state. Moving toward the edge, panels (*A1*–*A4*) of Fig. 2 track the OES at successive density extrema. At the maxima (*A1* and *A2*) the low-lying levels follow the sequences (1,1,3,⋯) and (1,2,4,⋯) that signal the I and *χ* anyon sectors of the APf phase (60). Strikingly, at the minima (*A3* and *A4*) the chirality appears reversed, though the counting is ambiguous. In the remainder of the paper we will argue these oscillations may be interpreted as alternating Pf and APf stripes, whose edges hybridize at low energy. Because the Pf and APf have different Hall viscosities, an interface between them is expected to carry a quantized dipole moment in proportion to the mismatch (61). Thus alternating Pf and APf stripes are expected to produce charge-density oscillations at the same $\lambda ∼5\ell_{B}$ period (57, 58).

In the ground state data just discussed, however, there is no obvious identification of the reversed OES as the Pf. Further evidence for such an interpretation emerges from low-lying excited states with a momentum boost relative to the ground state. In Fig. 2*B*, we show one such state in the $\delta k_{x}\approx -15.5\times 2\pi /L_{x}$ sector and energy $\delta E\approx 0.15E_{C}$ above the ground state. For technical reasons related to our DMRG implementation, the total charge also differs by δQ=1.5e. The density oscillations look very similar to those of the ground state, but with somewhat increased amplitude. Most remarkably, the low-lying OES now shows not just reversed chirality, but striking resemblance to either the Pf or APf OES in perfect correspondence with the density oscillations. We refer to *SI Appendix* for the OES of various other states in the low-energy manifold.

To summarize: in both the ground state and momentum-boosted state the OES oscillates in tandem with the charge density, but only in the large momentum state does the OES clearly correspond to alternating stripes of APf / Pf. To explain why, we propose a two-step mechanism. At a higher energy scale, the edge breaks into narrow stripes that alternate between Pf and APf phases, whose interface carries gapless Majorana modes. At a lower energy scale, these neighboring Majorana modes overlap and hybridize, which adds counterpropagating branches to the OES and hides the simple Pf counting. When a finite momentum boost is applied, we will show that Pauli exclusion turns off the low-*k* part of the hybridization. As a result, the Pf counting reappears in the low-*k* part of the boosted excited-state OES.

Although the behavior described above is robust across a broad range of $V_{L}$, $V_{R}$, and *d*, it is not universal. As shown in *SI Appendix*, much softer edges obtained by setting $\mu_{L}=-0.1E_{C}$ also display density oscillations (albeit of reduced magnitude), yet the OES follows Pf counting throughout the $L_{e}\approx 40\ell_{B}$ edge region before crossing over to APf in the bulk. This behavior fits within the same two-step framework: Pf and APf stripes first nucleate at the edge, and complete hybridization of their interface modes can yield an extended edge region with either Pf, APf, or PHPf order, depending on microscopic details. Because the hybridization depends sensitively on edge sharpness, interaction range, and excitation content, we observe several distinct patterns under different confinement profiles *SI Appendix*. The next sections develop an analytic framework for this hybridization, clarifying the range of possible edge structures, which we can then use to interpret the DMRG data.

We have demonstrated significant edge reconstruction for interfaces between $\nu =\frac{5}{2}$ and IQHE states. By contrast, interfaces between two incompressible $\nu =\frac{5}{2}$ states, which may form between large Pf and APf domains in the puddle picture of ref. 34, have not shown a similar form of reconstruction (58, 62). As such, we stress that the Majorana hybridization physics we discuss next is only expected to apply to the edges of the system, and not to any hypothetical internal domain walls.

## 3. Majorana Edge Hybridization

By comparing the Lagrangians $L_{\text{Pf}}$, $L_{\text{APf}}$, it is clear that an isolated interface between Pf and APf is generically described by four copropagating Majorana modes, $L_{\text{M}}^{\pm 4}$, with a negative or positive sign for Pf/APf or APf/Pf interface, respectively. The width of an isolated Pf/APf interface *w* is of the same order as the size λ/2 of the strips in our system (58), so we generically expect the Majorana modes from adjacent interfaces (which counter-propagate) to interact and gap each other out—an effect that is challenging to fully capture via DMRG due to the small energy scales involved. To address the reconstruction, we therefore take the stripe pattern from DMRG as an input, and study the hybridization of the resulting low-energy modes analytically, within an effective model. We model a striped edge parallel to the *x*-axis by a series of *N* strips alternating between Pf and APf extending from the vacuum at *y* = 0 to the bulk uniform QH liquid at $y=L_{e}=\left(N/2\right)\lambda$. (Two strips comprise a full oscillation, and for Fig. 2*B*, $N∼16,\lambda ∼5\ell_{B}$.) We take the strip adjacent to vacuum to be Pf, so that the bulk is APf for *N* odd, and Pf for *N* even (our results can readily be adapted to when the first strip is APf, but we make this choice for simplicity of exposition). Moving to a Hamiltonian description and ignoring disorder, the Majoranas are described by $H=H_{0}+H_{4N}+H_{c}+H_{c0}$, where

[4] $$\begin{array}{cc} H_{0} & =\sum_{k>0}\psi_{0}\left(-k\right)\left(v_{0}k\right)\psi_{0}\left(k\right),\;\text{and}\\ H_{4N} & =\sum_{k>0}\sum_{\alpha =1}^{N}\sum_{l,l^{\prime }=1}^{4}\psi_{\alpha ,l}\left(-k\right)\left[{\left(-1\right)}^{\alpha }v_{ll^{\prime }}k+if_{ll^{\prime }}^{\alpha }\right]\psi_{\alpha ,l}\left(k\right) \end{array}$$

respectively represent the single chiral mode at the *y* = 0 vacuum–Pf interface and the *N* quadruplets of modes at the Pf/APf interfaces, and

[5] $$\begin{array}{cc} H_{c} & =i\sum_{k>0}\sum_{\alpha ,\alpha^{\prime }=1}^{N}\sum_{l,l^{\prime }=1}^{4}\psi_{\alpha ,l}\left(-k\right)g_{l,l^{\prime }}^{\alpha ,\alpha^{\prime }}\psi_{\alpha^{\prime },l^{\prime }}\left(k\right)\;\text{and}\\ H_{c0} & =i\sum_{k>0}\sum_{\alpha =1}^{N}\sum_{l=1}^{4}g_{l}^{0,\alpha }\left[\psi_{0}\left(-k\right)\psi_{\alpha ,l}\left(k\right)-\psi_{\alpha ,l}\left(-k\right)\psi_{0}\left(k\right)\right] \end{array}$$

[6]
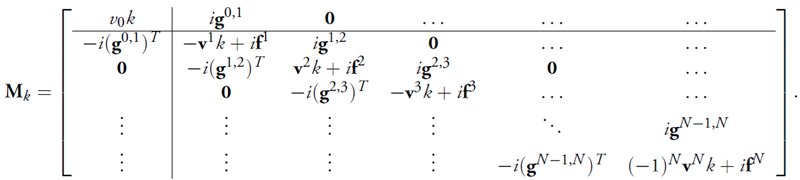


describe the couplings between the different Pf/APf interfaces and between the interfaces and the vacuum edge, with $g_{l,l^{\prime }}^{\alpha ,\alpha^{\prime }}=-g_{l^{\prime },l}^{\alpha^{\prime },\alpha }$. Next, we assume that all couplings except those between adjacent interfaces are negligible, so that only $g_{ll^{\prime }}^{\alpha ,\alpha \pm 1}$ and $g_{l}^{0,1}$ are nonzero (this is physically reasonable for local interactions; see Fig. 1). We then have $H=\sum_{k>0}\psi_{-k}M_{k}\psi_{k}$, where $\psi_{k}=\left(\psi_{0}\left(k\right),\psi_{1,1}\left(k\right),\psi_{1,2}\left(k\right),\ldots ,\psi_{N,4}\left(k\right)\right)$ and $M_{k}$ is of the form in Eq. **6**. Note that *H* is purely quadratic in Majoranas and we ignore higher-order terms as these are RG subleading relative to the terms we keep.

At this stage, we could in principle simply diagonalize *H* (numerically, if necessary) to determine the fate of the edge; however, since there are no clear symmetries and *H* has a very large number (∼16*N*) of independent parameters, this offers limited insight into the physics. As the Majorana hybridization model is inferred from the oscillating chirality of the OES, rather than quantitatively derived from the microscopic wavefunction, there is no clear mapping between the microscopic Hamiltonian and these parameters, forcing us to set them otherwise. One approach, that we turn to momentarily, is to simply choose parameters randomly, possibly with some additional structure e.g. reflecting the spatial falloff of couplings. To motivate our approach and to build intuition, we consider an initial toy model with a hierarchy of scales of the form $|g_{\left(1\;\text{or}\;2),(3\;\text{or}\;4\right)}^{\alpha ,\alpha +1}\left|∼\right|f_{\left(1\;\text{or}\;2),(1\;\text{or}\;2\right)}^{\alpha }\left|∼\right|f_{\left(3\;\text{or}\;4),(3\;\text{or}\;4\right)}^{\alpha }|,$ with all other couplings in f and g negligible relative to these. In this simplified limit, is easiest to first ignore $H_{c0}$ and work out what happens to $H_{4N}+H_{c}$ (the large lower-right block in Eq. **6**): the modes 3,4 for each interface α∈{1,2,…N−1} gap out against the counterpropagating modes 1,2 from interface *α* + 1, leaving gapless the pair of upstream modes 1,2 from *α* = 1 and the pair of modes 3,4 from *α* = *N* as shown in Fig. 1 (note that these are downstream/upstream for *N* even/odd). Crucially, these two pairs of modes are separated by the mesoscopic scale $L_{e}$; thus, when we now add back in $H_{c0}$, one of the modes in the first pair gaps out the downstream mode from *H*_0_, leaving a single upstream mode. In contrast, the “deep” modes remain gapless and decoupled from the edge. Evidently, independent of the bulk phase the physics near the edge is that of a charge mode and one upstream Majorana, so that it is described by $L_{c}+L_{\text{M}}^{-1}=L_{\text{PH}}$, the PH-Pfaffian edge theory. The bulk-boundary correspondence required by the Pf/APf phase in the far interior is restored by the two “deep” modes, with $L_{\text{M}}^{\pm 2}$, which propagate in precisely the direction such that they combine with the PH-like physical edge to yield $L_{\text{Pf/APf}}$ for the composite edge.

To see why this result is robust, observe that $H_{4N}+H_{c}$ can be viewed as a Bogoliubov-de Gennes Hamiltonian written in its Majorana representation; since there is no global symmetry, this lies in Class D of the “tenfold way” classification. The relevant integer topological invariant C, counts the net number of chiral Majorana modes remaining near the *α* = 0 interface beyond those required by the Pf/APf itself and can be related to a Chern number in a periodic system (which requires *N* even). The first step of our calculation above establishes that our toy model has C=2 (in a convention where the Majorana sectors of the Pf/APf have $C_{0}=-1,3$ and $|C_{0}+C|$ Majorana modes are localized close to the edge); the second step then follows upon coupling $H_{4N}+H_{c}$ to *H*_0_. Since C is an integer, it cannot change continuously, and hence is stable to small changes in parameters. Thus, the reconstruction represents a phase of the composite edge and hence is not fine-tuned.

We can generalize the above construction by considering random couplings between the modes (but still translationally-invariant parallel to the edge), chosen via

[7] $$\begin{array}{c} f_{ll^{\prime }}^{\alpha }=N\left[0,R^{|l-l^{\prime }|-1}\right],\;g_{ll^{\prime }}^{\alpha ,\alpha +1}=N\left[0,R^{|l-l^{\prime }+s-3|}\right], \end{array}$$

where $N[\mu ,\sigma^{2}]$ denotes a Gaussian random variable with mean *μ* and variance $\sigma^{2}$ (sampled independently for each matrix element), and the exponent of R∈[0,1] captures the effect of the spatial positions of modes on their couplings. The “offset” *s* controls the spatial overlap of the modes, so that mode 1 from interface *α* + 1 is closest to mode (4−s) of interface *α*, see Table 1. As flagged above, the width of an isolated interface is similar to the size of the strips in our system, making it unclear what values of *s* and *R* apply. This leads us to consider all possibilities. The scale hierarchy chosen in the simplified discussion above corresponds to taking *R* ≪ 1, and *s* = 1, but we can consider the problem more generally by drawing random instances of $H_{4N}+H_{c}$ with matrix elements distributed according to Eq. **7**, and computing C for each. Determining the relative probability of phases with different C under such sampling gives a rough sense of their extent in parameter space. (Note that the assumption of independently distributed random couplings may not be strictly correct for physical edge Hamiltonians, and so these probabilities should not be interpreted literally but more in the spirit of random matrix theory, as lending qualitative insight. For a given microscopic Hamiltonian, the values $f^{\alpha },g^{\alpha ,\alpha +1}$ are in principle fixed deterministically in the ground state. The random treatment is forced by our ignorance of the exact details of this map between microscopic Hamiltonians and the effective model.) The results (Table 1) reveal that there is always a finite window of C=2, which is required for the physical edge to emulate the PH-Pfaffian without fine-tuning. The phase structure is relatively insensitive to *R*; indeed, the finite C=2 window persists even for *R* = 1, when *s* plays no role and there is no structure beyond the nearest-neighbor form in Eq. **6**.

**Table 1.** Stability of reconstruction

		Probability of C=2 phase		
Mode offset		*R* = 0	*R* = 0.5	*R* = 1
*s* = 0	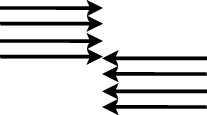	0.30	0.38	0.37
*s* = 1	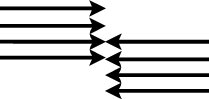	0.81	0.45	
*s* = 2	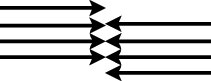	0.35	0.36	

Probabilities correspond to the frequency of C=2 for different *R* and *s* in Eq. **7**. The C=2 phase appears with a finite probability for all parameterizations. Statistical uncertainties are ∼0.01. The simplified case is one of the instances with R=0,s=1. For *s* negative, the distribution is similar to *s* = 0.

Above, we introduced randomness to sample the parameter space of a disorder-free system. Such a translationally invariant edge is an idealization of the physical system, but it highlights the key physics. Separately, we have also studied a problem with quenched disorder in the intermode couplings (i.e, we write Eq. **6** in position (*x*) space with spatially varying random $f^{\alpha }\left(x\right),g^{\alpha ,\alpha^{\prime }}\left(x\right)$), and verified that here too there is a finite window where $H_{4N}+H_{c}$ hosts a pair of chiral modes at the physical edge and the deep interior and hence effectively has C=2 *SI Appendix*.

We emphasize that this observation of a stable phase of edge reconstruction in agreement with experiment is a key theoretical advance which is to be contrasted with previous constructions (34). While both constructions rely on a mixing between Pf and APf to produce a PH-Pf response, the bulk picture of ref. 34 is not as robust as our edge construction. For bulk puddles, only fine-tuned values of effective model parameters yield the correct conductance, and the probability of obtaining such a conductance within a random matrix theory treatment in the spirit of Table 1 would be zero. While our construction does not guarantee ${\overset{¯}{\kappa }}_{\mathrm{xy}}=1/2$, it provides a robust mechanism for it.

## 4. Interpretation of DMRG Results

With this theoretical picture in mind, we now return to the interpretation of the DMRG results. First recall that for smoother edges ($\mu_{L}=-0.1E_{C}$), DMRG still finds density oscillations, but the OES is consistent with the formation of a large $L_{e}∼40\ell_{B}$ stripe of Pfaffian at the edge. Such behavior implies that the four Majorana modes bound to successive Pf–APf interfaces have strongly hybridized into a pair of counterpropagating channels, thereby realizing the C=4 edge configuration introduced above. Indeed, we must emphasize that our proposal does not require the formation of a C=2 PHPf boundary, it simply allows for it to be generic; consistent with this, the random matrix results show a probability for C=2 which is large but less than 1. We do not currently have a microscopic understanding of how the edge sharpness relates to the f,g parameters of the effective model, though this would be useful to explore in future work in order to understand how experiments might tune between different regimes.

Second, we return to our observation that when DMRG is used to target excited states in a boosted $k_{x}$ sector, the OES shows clear oscillations between Pf and APf, while the ground state does not. To understand this, consider a much simpler, but analogous, scenario in which a strip of p+ip superconductor forms in a trivial *ν* = 0 background, resulting in left/right moving chiral Majorana modes $\psi_{L/R}$ at the boundaries of the strip. In the absence of hybridization between them, an entanglement cut in the middle of the p+ip strip would look like that of p+ip SC, e.g. right-handed chiral. Hybridization between the edges modifies the entanglement, resulting in an entanglement spectrum with left and right moving modes (of different velocity) with zero net chirality. However, we will show that in states with boosted momentum, this hybridization entanglement is removed from the low-lying entanglement spectrum: the low-lying spectrum is thus expected to look like the p+ip SC in the boosted-momentum state. An analogous calculation applies to the multichannel case.

To make this calculation concrete, consider left and right moving Majorana modes, $\psi_{L}\left(k\right),\psi_{R}\left(k\right)$, satisfying $\left\{\psi_{\mu }\left(k\right),\psi_{\nu }\left(k^{\prime }\right)\right\}=\delta_{\mu \nu }\delta_{k+k^{\prime }}$. A model for their hybridization is

[8] $$\begin{array}{cc} H=\frac{1}{2}\sum_{k} & \left[\psi_{R}\left(-k\right)\left(vk\right)\psi_{R}\left(k\right)+\psi_{L}\left(-k\right)\left(-vk\right)\psi_{L}\left(k\right)\right.\\ & \left.+2ig\psi_{R}\left(-k\right)\psi_{L}\left(k\right)+\cdots \right] \end{array}$$

When *g* = 0, the vacuum ${|0\rangle }_{L}{|0\rangle }_{R}$ is defined by the property $\psi_{R}\left(k\right){|0\rangle }_{R}=\psi_{L}\left(-k\right){|0\rangle }_{L}=0$ for *k* > 0. Thus $\psi_{R}\left(-k\right),\psi_{L}\left(k\right)$ for *k* > 0 act like creation operators on top of this vacuum.

Following an analogous calculation in ref. 63, the hybridized ground state takes the form

[9] $$|g\rangle =\prod_{k>0}\left[\sqrt{1-p_{k}}+i\sqrt{p_{k}}\psi_{R}(-k)\psi_{L}(k)\right]|0\rangle_{L}|0\rangle_{R},$$

where ${|0\rangle }_{L/R}$ is the vacuum in the absence of hybridization and $p_{k}=\frac{1}{2}-\frac{\mathrm{vk}}{2|g|}+O\left(k^{3}\right)$. Eq. **9** is already in Schmidt form, with single-particle entanglement energies determined by $e^{-\epsilon_{k}}=p_{k}/\left(1-p_{k}\right)$, giving $\epsilon_{k}=2vk/\left|g\right|+\cdots$.

This is not the entirety of the entanglement, however, as we must remember that the “vacuum” ${|0\rangle }_{L}{|0\rangle }_{R}$ is a p+ip SC, which itself has a chiral entanglement spectrum $\epsilon_{k}^{\left(0\right)}=-v_{B}k$. Keeping track of the signs, its entanglement chirality is opposite to the hybridization entanglement just discussed. The full entanglement spectrum therefore contains two branches: the hybridized branch $\epsilon_{k}$ arising from the coupling between the left and right modes, and the intrinsic branch $\epsilon_{k}^{\left(0\right)}$ inherited from the p+ip bulk. The hybridized branch $\epsilon_{k}$ can obscure the counting of the intrinsic branch $\epsilon_{k}^{\left(0\right)}$ when the two sets of levels overlap.

Now instead consider the state with momentum ΔK larger than the ground state. For a monotonic dispersion relation, this is obtained by filling the lowest *k*-state excitations up to some $k_{*}$, $\Delta K=\sum_{k<k_{*}}k$. The resulting lowest-energy state is verified to be

[10] $$\begin{array}{c} \begin{array}{c} |g,\Delta K\rangle =\prod_{k>k_{*}}\left(\sqrt{1-p_{k}}+i\sqrt{p_{k}}\psi_{R}\left(-k\right)\psi_{L}\left(k\right)\right)\\ \times \prod_{k<k_{*}}\psi_{R}\left(-k\right){|0\rangle }_{L}{|0\rangle }_{R}. \end{array} \end{array}$$

The key observation is that the modes $k<k_{*}$ no longer contribute entanglement, since there is no superposition in these sectors. This can alternatively be understood by noting that in the boosted state, both the L and R *k*-modes are occupied for $k<k_{*}$, so their hybridization is Pauli blocked. The lowest entanglement levels $\epsilon_{k}$ for $k<k_{*}$ are thus removed from the entanglement spectrum (equivalently, $\epsilon_{k}\to \infty$ for $k<k_{*}$).

Boosting to large momentum thus removes the low-lying entanglement that would otherwise come from hybridization, effectively recovering the unhybridized result. This mechanism generalizes to the multichannel case relevant to the Pf-APf domains. In the boosted ΔK>0 states, the low-*k*$\psi_{R,\alpha }\left(k\right)$ modes (where *α* now runs over wires and flavors) become filled, so that their hybridization with the L modes is Pauli blocked. We conjecture that this mechanism results in the difference in OES shown in Fig. 2 *A* and *B*, where the boosted state shows an OES that oscillates between Pf and APf, as if the edges are unhybridized.

## 5. Equilibration and Edge Transport

We have established that for a range of parameters, there can be a mesoscopic separation of the “topologically mandated” Pf/APf edge into a PH-Pfaffian like physical edge described by $L_{c}\left[\phi \right]+L_{M}^{-1}\left[\chi \right]$ and a pair of deep chiral neutral modes $L_{M}^{\pm 2}\left[\psi_{1},\psi_{2}\right]$. In order for this structure to explain experiment, it is crucial that these two spatially separated modes remain decoupled even in the presence of generic interactions $L_{\text{int}}$ and/or disorder $L_{\text{dis}}$. The most relevant couplings between $L_{c}\left[\phi \right]+L_{M}^{-1}\left[\chi \right]$ and $L_{M}^{\pm 2}\left[\psi_{1},\psi_{2}\right]$ in the RG sense are terms $∼\chi \psi_{1,2}$. But first, as $\psi_{1,2}$ are close together, it is natural to expect a term $im\psi_{1}\psi_{2}\subset L_{\text{int}}$. Due to the separation of interfaces, we expect this term to have a larger prefactor than any $\chi \psi_{1,2}$ term in $L_{\text{int}}+L_{\text{dis}}$; therefore, we first eliminate $\psi_{1}\psi_{2}$ by rotating to a new set of decoupled Majoranas ${\tilde{\psi }}_{1,2}$, but at momenta $\pm k_{0}$, with $k_{0}∼O\left(\ell_{B}^{-1}\right)$ on dimensional grounds. The residual couplings of the form $\chi {\tilde{\psi }}_{1,2}$ can then be treated perturbatively. Already at the bare level, these are suppressed by two effects: 1) the small wavefunction overlap between the different edge modes and 2) the momentum mismatch between ${\tilde{\psi }}_{1,2}$ and the single Majorana mode *χ* which must remain at *k* = 0. The latter suppresses both interactions and disorder: in experiments, we expect the disorder potential is correlated over ∼100 $\text{nm}∼10\ell_{B}\gg k_{0}^{-1}$, making it too smooth to effectively supply the needed momentum boost. Thus, although $\chi {\tilde{\psi }}_{1,2}$ has scaling dimension Δ=1, and is hence RG relevant in both the clean and disordered cases (64), the RG flow for experimentally reasonable length scales and temperatures cannot overcome the two bare suppression effects, and so such terms cannot effectively equilibrate the physical edge with the deep modes *SI Appendix*. All other allowed terms coupling ϕ,χ to $\psi_{1,2}$ necessarily have higher scaling dimensions while still suffering from the same suppression effects so they can be ruled out as sources of equilibration as well. We argue in *SI Appendix* that these effects also prevent coupling of $\psi_{1,2}$ to the floating contacts used in experiments. This complete decoupling of the deep modes is crucial; note that, while lack of equilibration between modes is generally argued to increase ${\overset{¯}{\kappa }}_{\mathrm{xy}}$, this argument no longer applies when some subset of modes, beyond just being out of equilibrium with other modes, also further decouple from the contacts *SI Appendix*. Taken together, this means that the deep modes will not contribute to thermal Hall measurements. A complete theory of how this model couples to the contacts is an important direction for future work, as measuring how the thermal Hall conductance depends on the contact size and shape could be a key tool for experimentally testing our picture of the edge. In particular, we predict that with contacts extending sufficiently far over the sample, or for larger lengths of the edge between the contacts, the PH-Pf value ${\overset{¯}{\kappa }}_{\mathrm{xy}}=\frac{1}{2}$ should eventually move toward ${\overset{¯}{\kappa }}_{\mathrm{xy}}=\frac{3}{2}$ or ${\overset{¯}{\kappa }}_{\mathrm{xy}}=-\frac{1}{2}$, revealing the true bulk Pf or APf order.

Having established the decoupling of the inner theory $L_{M}^{\pm 2}\left[\psi_{1},\psi_{2}\right]$ from the system edge, the one remaining subtlety is the question of how the PHPf-like modes *ϕ* and *χ* in the edge theory $L_{c}\left[\phi \right]+L_{M}^{-1}\left[\chi \right]$ equilibrate. This question of PHPf edge equilibration has been addressed in ref. 64, which found that the most RG relevant equilibration operator is $O_{\text{PHPf}}=\left(\partial_{x}\phi \right)\chi \partial_{x}\chi$. But its relatively large scaling dimension Δ=3 makes it highly RG irrelevant, and perturbative calculations predict that this term would in practice be too weak to effectively equilibrate the edge. The question of how a PHPf edge equilibrates thus remains open, and any resolution will probably require a nonperturbative computation that likely involves terms not captured in the scaling limit of the edge theory. This problem is not unique to our construction, and is in fact also present in any picture involving a PHPf-like edge, including setups with a uniform PHPf bulk state.

## 6. Concluding Remarks

While our calculation was performed considering an interface between ν=5/2 and *ν* = 2 with either a Pf or APf bulk, the physics is essentially identical if we consider the interface between ν=5/2 and *ν* = 3—a PHPf edge can form next to the integer state, which is measured by any transport experiment, and two additional states are left deep in the bulk, decoupled from edge measurements. Thus, as long as we assume a sufficiently steep confining potential, our theory can be brought into agreement with the thermal transport and noise experiments (22–25), and consistency with the electronic probes (41–44), which do not sharply discriminate between the non-Abelian orders. In summary, we have demonstrated that it is possible to separate the bulk topological physics from the observed edge, and that for a range of reasonable parameters this separation can be made consistent with experiment.

How might this new edge structure affect Fabry–Perot interferometry measurements (e.g., ref. 65)? Since the charge modes nearest the edge must be partially reflected from the point contacts, it is reasonable to assume that the deep modes are fully reflected, thus forming a disconnected dot in the middle of the cavity. In this case, one should see interference of the PH-Pfaffian edge modes, irrespective of the “true” bulk order. Similar arguments apply to other possible edge structures for other choices of inter-Majorana coupling or edge confinement scale. The central lesson remains that the link between bulk and edge in the non-Abelian ν=5/2 QH liquid is more subtle than previously imagined, and is a richer problem than dictated by topological considerations alone.

## Supplementary Material

Appendix 01 (PDF)

## Data Availability

The code used and data generated for this study are available online at Github (66). All other data are included in the manuscript and/or *SI Appendix*.
