# Supplementary material for: Majorana edge reconstruction and the ν = 5/2 non-Abelian thermal Hall puzzle
Source: Proc Natl Acad Sci U S A. 2026 Nov 5;123(45):e2608514123. doi: 10.1073/pnas.2608514123 (PMC13752366; doi:10.1073/pnas.2608514123)
Supplement: Supplementary file 1 [file pnas.2608514123.sapp.pdf]

Supporting Information for

Majorana edge reconstruction and the $\nu = 5/2$ non-Abelian thermal Hall puzzle

Tevž Lotrič, Taige Wang, Michael P. Zaletel, Steven H. Simon and S. A. Parameswaran

Corresponding Author: Tevž Lotrič.
E-mail: tevz.lotric@physics.ox.ac.uk

This PDF file includes:

Supporting text
Figs. S1 to S7
SI References

Supporting Information Text

1. An overview of candidate states for the $\nu = \frac{5}{2}$ FQHE state

To understand the variety of quantum Hall states at filling $\frac{5}{2}$, we focus on the half-filled first Landau level, which contains N_e electrons in $N_\phi = \frac{1}{\nu}N_e = 2N_e$ flux quanta (with $\nu = 1/2$ the fractional filling fraction). The Pfaffian-like family of states may be understood in the composite fermion picture, where we imagine 2 flux quanta to be attached to each electron to form a composite fermion (CF). After flux attachment, the CFs see only the remaining field, $B^* = B(1-2\nu) = 0$, meaning that the CFs see no field on average. This can lead to a gapless Fermi liquid state of the CFs – the Composite Fermi Liquid (CFL). This is to be contrasted with many odd-denominator states, such as $\nu = \frac{1}{3}$, where composite fermions may fill an integer number of Landau levels, forming an insulating state.

While the gapless CFL can remain stable (and it does at $\nu = \frac{1}{2}$), it can also undergo various instabilities. An interesting class of such instabilities are pairing instabilities, in particular in the chiral triplet channels. For an odd integer l, the composite fermions may weakly pair with order parameter $\Delta(k) \sim (k_x + ik_y)^l$, leading to a gapped state (1). We remark that a paired state of composite fermions does not imply a paired state for the underlying electrons. Taking $l = -3, -1, 1$ yields the anti-Pfaffian, PH-Pfaffian and Pfaffian states discussed in the main text, respectively. The pairing channel is directly related to the number of chiral Majorana edge modes. All three states have a 6-fold topological ground state degeneracy on the torus. The non-Abelian sector of all three states respects Ising fusion rules $\sigma \times \sigma = 1 + \psi$, $\sigma \times \psi = \psi$, $\psi \times \psi = 1$, and the non-Abelian σ anyon, associated with a half-flux quantum, carries charge $e/4$. While the fusion rules are the same for all three orders, the anyon exchange phases differ (2, 3). It can be difficult to determine which channel is favored by using the composite Fermion picture, but numerical calculations favor the anti-Pfaffian or Pfaffian channels, as discussed in the main text (although other values of l not discussed above are all in principle allowed, they just seem to not be energetically favorable). Still, the fact that the composite fermion picture does not immediately yield a unique gapped ground state candidate can be thought of as an indicator of the close competition between candidate states, and the resulting difficulty in determining the state's true nature.

Another approach, removed from paired composite-fermion states, would be multi-component quantum Hall states, such as the abelian Halperin-331 or 113 states, which however do not support non-Abelian excitations, and also do not clearly give rise to a half-integer quantized thermal Hall response (2).

2. DMRG calculation

A. Setup. *Step 1—Bulk ground states.* We work on an infinitely long cylinder of circumference $L_x = 18\ell_B$. Metallic screening planes at $z = \pm d$ with $d = 5\ell_B$ render the Coulomb interaction short-ranged,

$$V_d(q) = \frac{2\pi}{q}\tanh(qd), \quad [1]$$

in units of $E_C = e^2/4\pi\varepsilon\ell_B$. Results for $d = 10\ell_B$ are qualitatively identical; we therefore quote data for $d = 5\ell_B$.

The Hamiltonian takes the form

$$H = \frac{1}{2}\int d^2\mathbf{r}d^2\mathbf{r}'\rho(\mathbf{r})V_d(\mathbf{r}-\mathbf{r}')\rho(\mathbf{r}'). \quad [2]$$

where $\rho(\mathbf{r})$ is the density operator projected to the $N = 1$ Landau level (LL). Neglecting LL mixing, the Moore–Read Pfaffian (Pf) and its particle–hole conjugate, the anti-Pfaffian (aPf) are exactly degenerate; we choose the aPf sector, the phase expected to prevail once LL mixing is reinstated.

Step 2—Chemical-potential calibration. The "defect"-DMRG protocol (4, 5) isolates individual anyons in a wide window whose left and right boundaries are pinned to topological sectors $[l, r]$. For the Pfaffian family $[0110, 1010]$ traps a charge $+e/4$ quasiparticle and $[0110, 0101]$ a charge $-e/4$ quasihole, yielding

$$E_{+e/4} = 0.54E_C, \qquad E_{-e/4} = -0.51E_C \quad [3]$$

for $L_x = 18\ell_B$ and $d = 5\ell_B$. The shared Madelung term cancels in the difference, fixing the mid-gap chemical potential to

$$\mu_{\mathrm{aPf}} = \frac{4}{2}\left(E_{+e/4} - E_{-e/4}\right) = 2.10E_C. \quad [4]$$

Step 3—Edge-reconstruction setup. A split top gate held at $V_L \neq V_R$ and a grounded bottom gate ($V_B = 0$) generate the step-like potential

$$U(y) = \frac{V_L}{2} + \frac{V_R - V_L}{\pi}\arctan(e^{\pi y/2d}), \quad [5]$$

where $y = 0$ sits at the interface and the smoothness is controlled by d. Target chemical potentials μ_L (vacuum) and μ_R (aPf) fix the gate voltages to $V_{L/R} = -2\mu_{L/R}$, with $\mu_L = -2E_C$ chosen inside the cyclotron gap and $\mu_R = 2.1E_C$.

Beginning with the homogeneous MPS tensors obtained in Step 1, we construct an inhomogeneous ansatz composed of three regions: a semi-infinite vacuum lead on the left, a semi-infinite aPf lead on the right, and a window of N_s variational tensors in between. The window is seeded with N_{vac} vacuum tensors followed by $N_{\mathrm{aPf}} = N_s - N_{\mathrm{vac}}$ aPf tensors.

Ideally one would minimize the energy in every sector labeled by the conserved quantum numbers—the charge Q and the total momentum K_x in the window. At finite bond dimension χ, however, the variational freedom afforded by a given seed

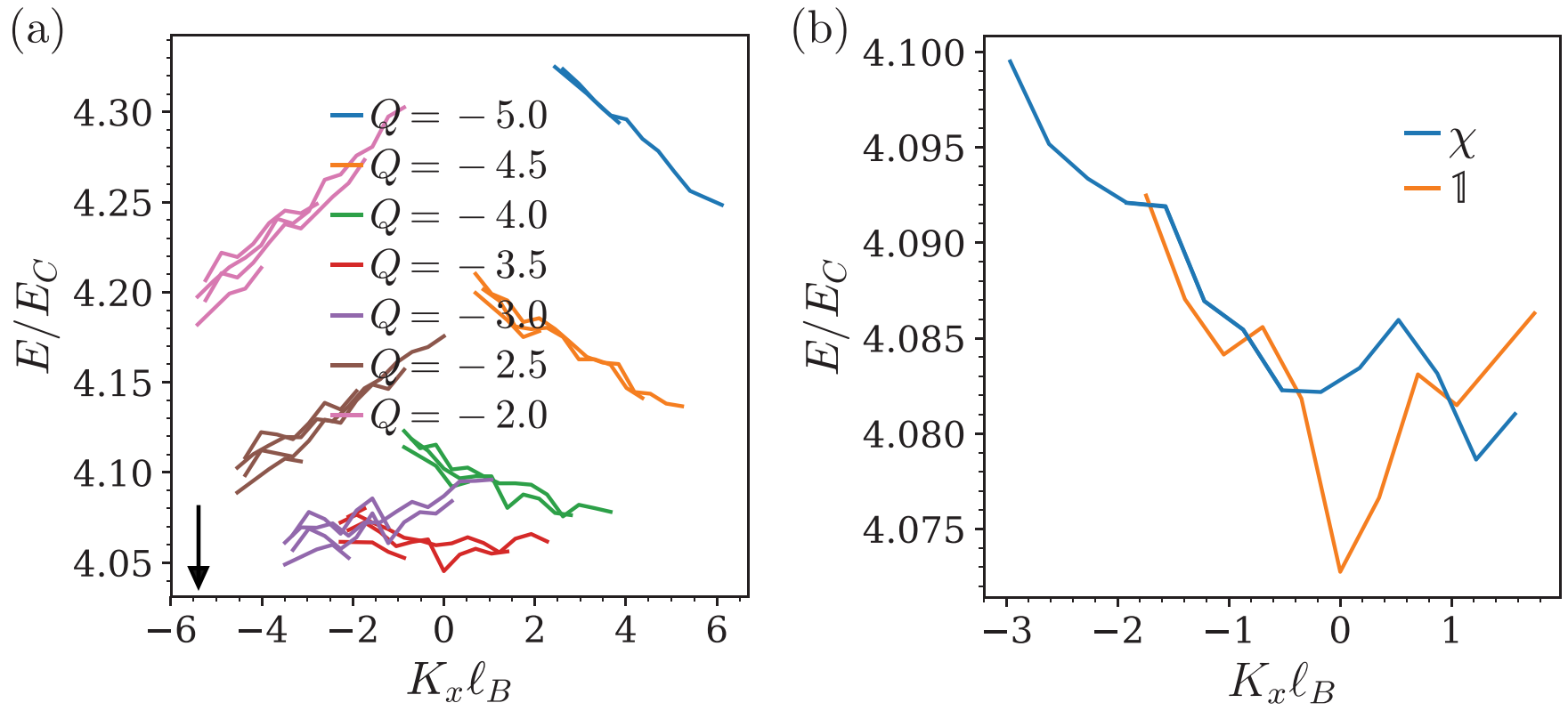

Fig. S1. Edge energy E as a function of total momentum K_x. (a) Comprehensive Q survey obtained with $N_s = 104$ and $\chi = 512$; colors distinguish different charge sectors. The black arrow indicates the momentum $K_x \approx -15.5 \times 2\pi/L_x$ of the state in Fig. 2(B). The collapse is not perfect due to limited N_s and χ. (b) Enlarged view of the optimal sector $Q = -3.5e$, calculated with $N_s = 360$ and $\chi = 2048$ where we see perfect collapse. Two topological branches are resolved: the trivial anyon sector $\mathbb{1}$ (orange) and the neutral-fermion sector χ (blue). Energies are quoted in units of E_C. The slight difference in energies between (a) and (b) is a result of using different N_s and χ.

characterized by N_{vac} is limited by modest fluctuations in (Q, K_x). We therefore sweep N_{vac}; each increment $N_{\text{vac}} \to N_{\text{vac}} + 1$ transfers charge $\Delta Q = -e/2$ and produces a correlated shift in K_x. In this way every charge sector is accessible, but for a fixed Q the set of reachable momentum K_x is still restricted at finite χ. For the relatively sharp confining potential considered here, the true ground state always falls within the accessible K_x set. By varying N_{vac}—and the allowed (Q, K_x) for each seed—we sweep each ansatz to convergence and retain the complete low-energy manifold.

B. Results. Fig. S1(a) compiles the lowest energy obtained in every (Q, K_x) sector encountered during an N_{vac} sweep carried out with a window $N_s = 104$ and bond dimension $\chi = 512$. We calibrate $Q = 0$ to the configuration that exactly fills the right half of the variable window and rigidly shift each dispersion so that the ground state sits at $K_x = 0$. The energetically preferred edge carries charge $Q = -3.5e$; this sector hosts both the ground state and the entire set of low-lying excitations analyzed below.

Curves sharing the same color correspond to different values of N_{vac}. In principle, they should collapse onto just two curves, because for a given (Q, K_x) the ground-state energy depends only on the boundary condition. The imperfect collapse in Fig. S1(a) originates from the limited window size N_s and bond dimension χ. Enlarging the window to $N_s = 360$ and increasing the bond dimension to $\chi = 2048$ in the $Q = -3.5e$ sector restores perfect overlap among the curves for different N_{vac}. In both cases, we perform the calculation with the left boundary in both the 0110 and the 1001 sector. These two choices differ by a neutral fermion threading the window and give rise to the pair of dispersion branches shown in Fig. S1(b). The lower branch is therefore assigned to the trivial sector $\mathbb{1}$, while the higher branch corresponds to the neutral-fermion sector χ. Their splitting at $K_x = 0$ is below $5 \times 10^{-3} E_C$ and decreases further as χ is increased, consistent with neutral-fermion modes that remain gapless in the thermodynamic limit.

The ground state supports a charge-density wave with period $d \approx 5\ell_B$ and decay length $L_e \approx 40\ell_B$ (main-text Fig. 2A). To expose its topological structure we evaluate the orbital entanglement spectrum (OES) at *every* bond of the $\chi = 2048$ ground-state MPS; the complete data set is displayed in Fig. S2. Deep in the bulk the counting is anti-chiral with sequences $(1, 1, 3, \dots)$ or $(1, 2, 4, \dots)$, reflecting the two aPf anyon sectors that the cut can select. *At each density maximum* the counting remains anti-chiral aPf counting, whereas *at each density minimum* it becomes chiral–though it does not match the Pf pattern because local hybridization between counter-propagating modes distorts edge modes along entanglement cuts. This alternation persists for three full oscillations before the OES reverts to uniform aPf, providing further support for the picture of a parent configuration of alternating Pf and aPf strips.

Additional insight comes from an excited state in the $Q = -2e$ sector (main-text Fig. 2B). Its OES, plotted bond-by-bond in Fig. S3(b), shows unambiguous chiral Pf counting at every density minimum, while the maxima retain the anti-chiral aPf structure. Although this state is higher in energy by $\Delta = 0.15E_C$, the clean one-to-one correspondence between Pf/aPf counting and density minima/maxima allows us to interpret the *unhybridized parent* to be alternating Pf–aPf strips: the ground state is obtained from the same Pf–aPf strip pattern after partial Majorana-mode hybridization. The stronger and longer-range density oscillation is consistent with the larger excess charge ($\sim 1.5e$) accommodated at the edge.

3. Intermediate confinement and $\mathcal{C} \neq 2$ phases

To explore an intermediate confining potential, we set the vacuum-side chemical potential to $\mu_L = -0.1E_C$ while keeping $L_x = 18\ell_B$ and $d = 5\ell_B$ unchanged. The resulting ground state, shown in Fig. S4, exhibits a single Pf strip roughly $40\ell_B$ wide at the edge. Across this strip the orbital entanglement spectrum is uniformly chiral with Pf counting $(1, 1, 3, \dots)$ or $(1, 2, 4, \dots)$ depending on the entanglement cut, independent of whether the local charge density sits at a maximum or a minimum–evidence

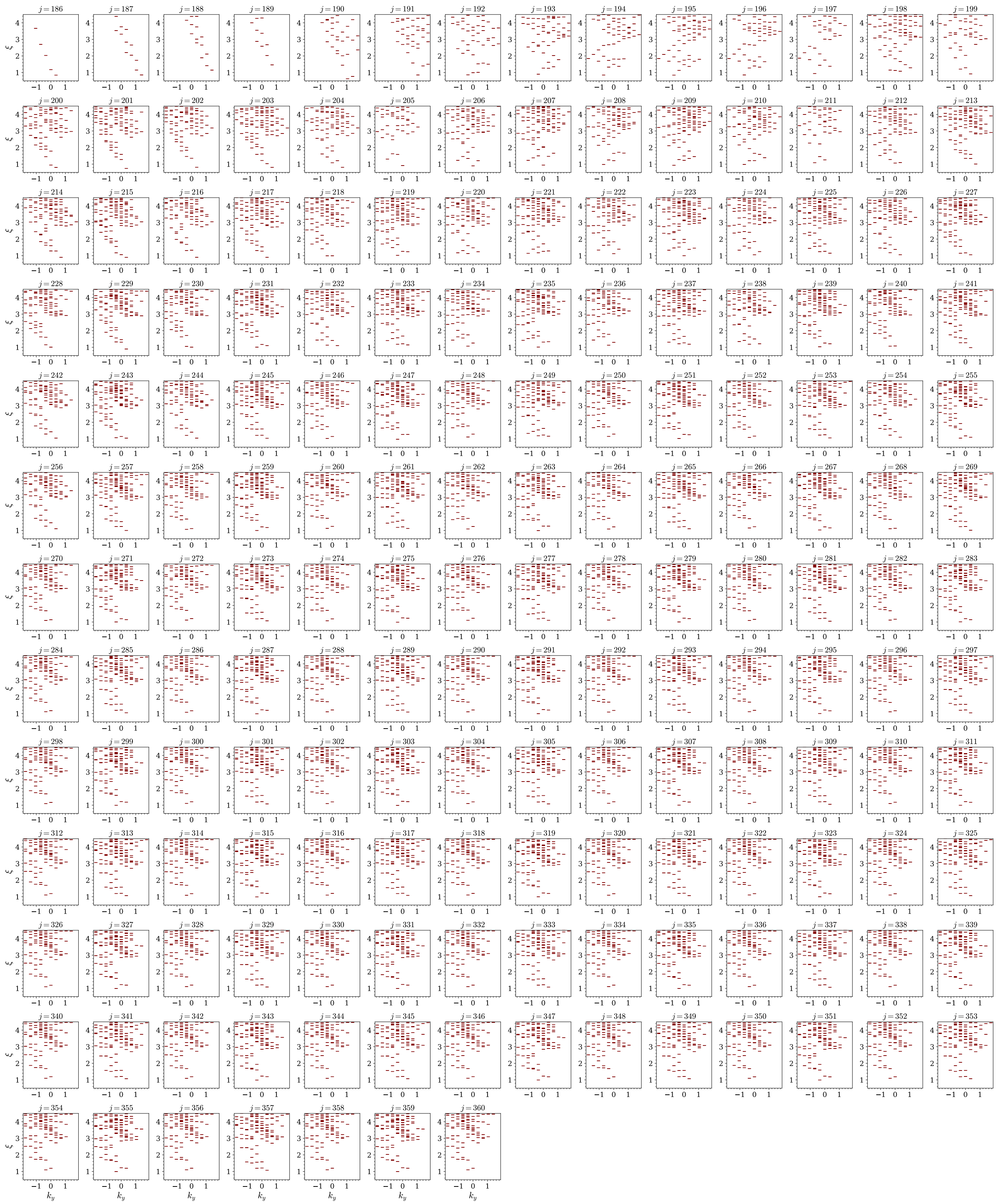

Fig. S2. Entanglement spectra at every bond j of the $\chi = 2048$ ground state shown in main-text Fig. 2(A). Anti-chiral aPf counting at density maxima alternates with chiral counting at density minima.

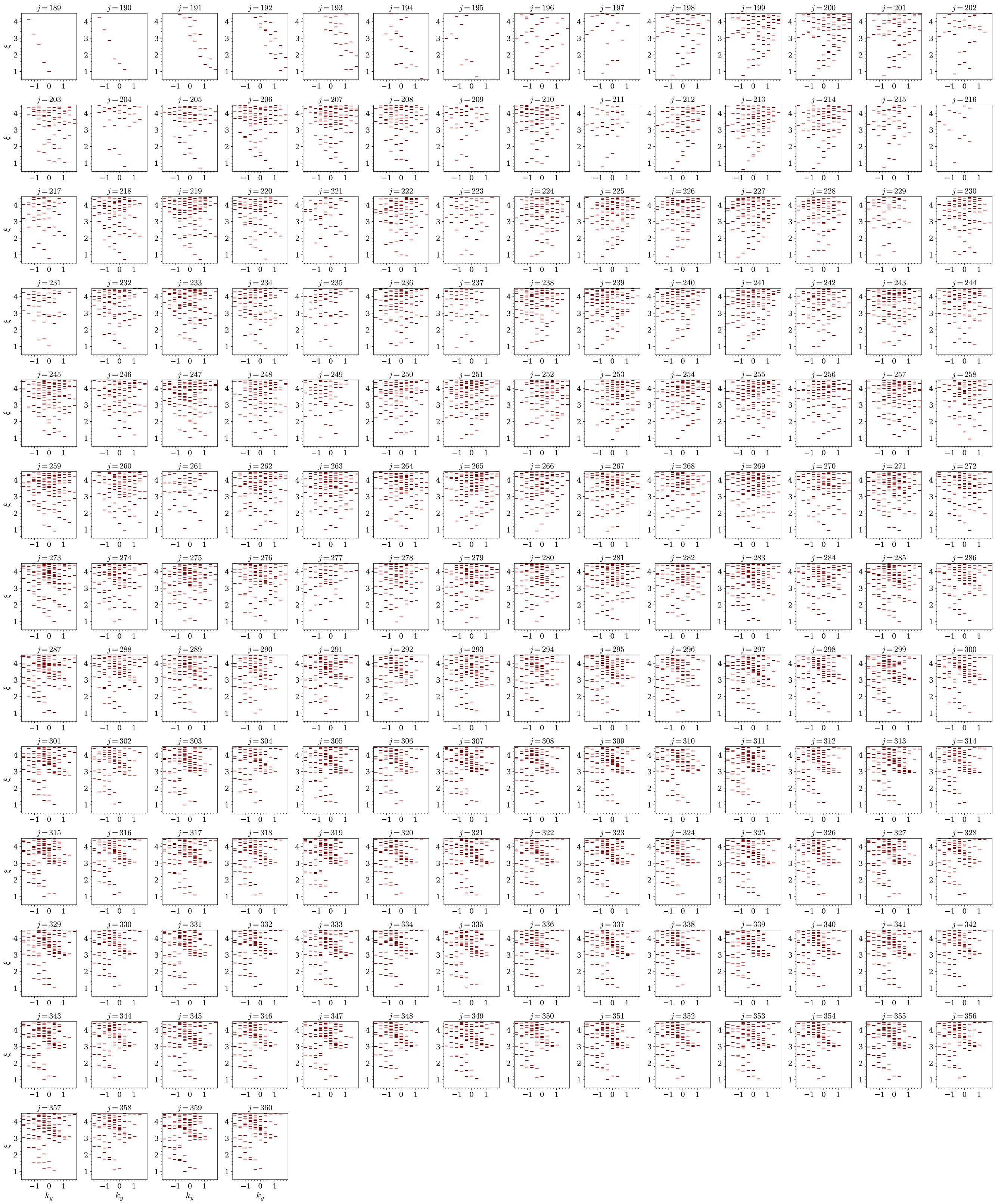

Fig. S3. Entanglement spectra at every bond j of the $\chi = 2048$ excited state shown in main-text Fig. 2(B). Density minima shows clear Pf counting.

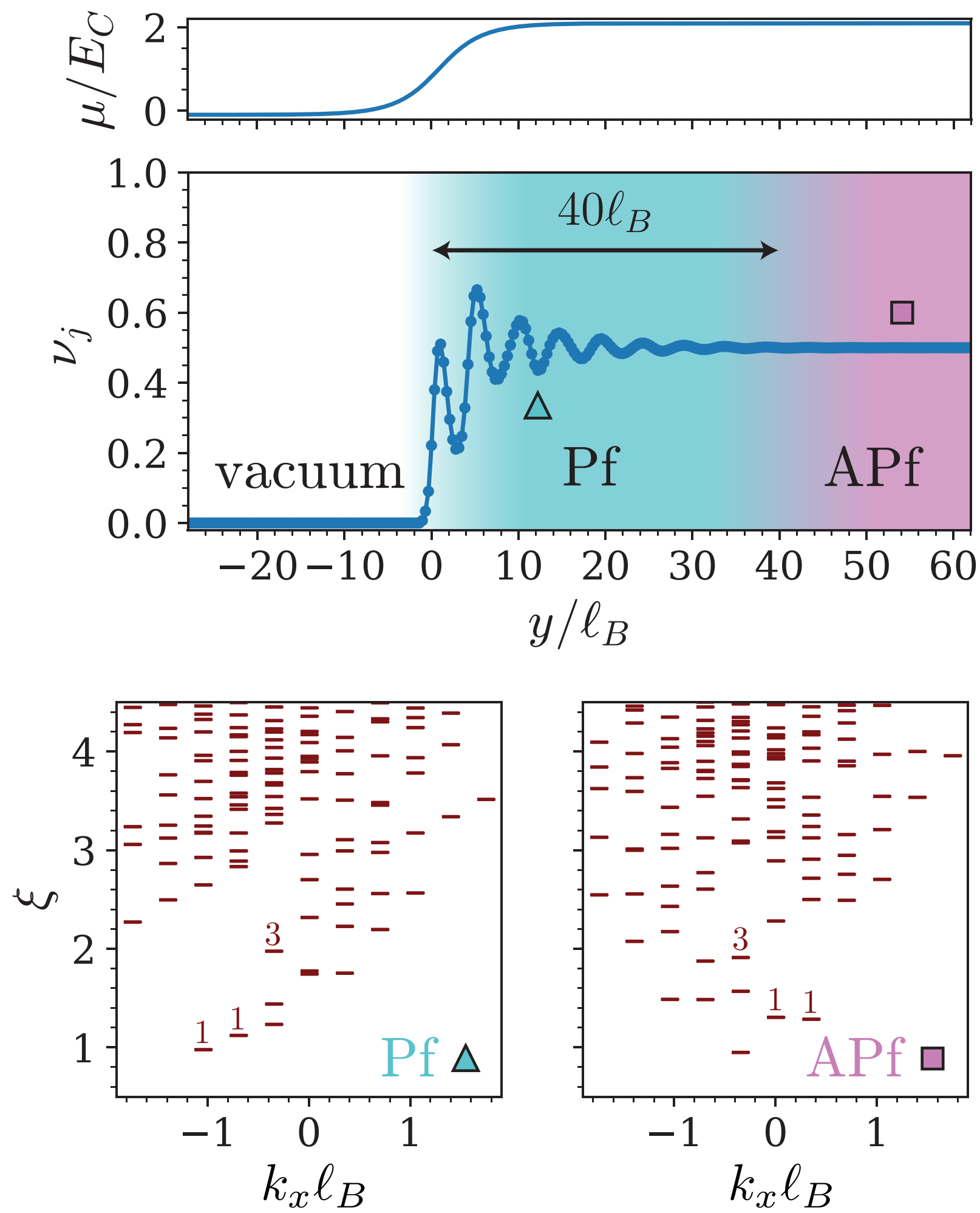

Fig. S4. Edge Reconstruction for an Intermediate Confining Potential. (a) Background chemical potential $\mu(y)$ used in the DMRG calculation; the gentle slope corresponds to $\mu_L = -0.1E_C$ on the vacuum side. (b) Orbital filling factor ν_j obtained for the ground state. The shaded region highlights a single Pf strip of width $\sim 40\ell_B$ that forms between the vacuum and the aPf bulk. (c,d) Orbital entanglement spectra (OES) taken at the points marked by the △ (Pf strip) and □ (aPf bulk) in panel (b). Panel (c) shows uniformly chiral Pf counting $(1, 2, 4, \dots)$, while panel (d) displays the anti-chiral aPf counting $(1, 1, 3, \dots)$, confirming complete hybridization of the counter-propagating Majorana modes inside the Pf strip. The charge-density oscillation retains its characteristic spatial period $\approx 5\ell_B$ but with reduced amplitude relative to the sharper-edge case. Data were obtained on a cylinder of circumference $L_x = 18\ell_B$ with gate spacing $d = 5\ell_B$, $\mu_R = 2.1E_C$, bond dimension $\chi = 2048$, and $N_s = 360$.

that the counter-propagating Majorana modes have hybridized completely. The charge-density wave retains its characteristic period $\approx 5\ell_B$ but its amplitude is noticeably reduced, consistent with the gentler potential gradient.

A smoothly varying confinement corresponds, in the coupled-stripe picture of Sec. IV, to stronger tunneling between Majorana modes that are separated by an aPf domain ($\mathbf{g}^{1,2}, \mathbf{g}^{3,4}, \ldots$) than between those separated by a Pf domain ($\mathbf{g}^{2,3}, \mathbf{g}^{4,5}, \ldots$); cf. Eq. 5. Departing from the balanced distribution assumed in Eq. 7 therefore favors the $\mathcal{C} = 4$ phase over the $\mathcal{C} = 2$ phase discussed in the main text. In the extreme limit where $\mathbf{g}^{1,2} \gg \mathbf{g}^{2,3}$ the model yields a fully hybridized Pf strip, exactly as observed numerically in Fig. S4. Although the entanglement spectrum no longer resolves individual Pf/aPf domains, the prevalence of chiral Pf counting throughout the oscillatory region is fully consistent with this $\mathcal{C} = 4$ limit of the stripe construction.

4. Bloch Hamiltonian and topological invariants

This section details the calculation of the topological invariant $\mathcal{C}$ within the $H_{4N} + H_c$ part of our model. We start by assuming N is even, in which case the invariant may be interpreted as a Chern number. We enforce a periodic structure with a unit cell of 8 Majorana modes by requiring $\mathbf{v}^\alpha = \mathbf{v}^{\alpha+2}$, $\mathbf{f}^\alpha = \mathbf{f}^{\alpha+2}$ and $\mathbf{g}^{\alpha,\alpha+1} = \mathbf{g}^{\alpha+2,\alpha+3}$. Crucially, we allow for $\mathbf{f}^1 \neq \mathbf{f}^2, \ldots$ – there is no symmetry to enforce this. After a Fourier transformation perpendicular to the edge, we get the 8×8 Bloch Hamiltonian

$$H(k,q) = \begin{bmatrix} -\mathbf{v}^1 k + i\mathbf{f}^1 & i\mathbf{g}^{1,2} - i(\mathbf{g}^{2,3})^T e^{-iq} \\ -i(\mathbf{g}^{1,2})^T + i\mathbf{g}^{2,3} e^{iq} & \mathbf{v}^2 k + i\mathbf{f}^2 \end{bmatrix}. \quad [6]$$

While Eq. 6 obeys $H(k,q) = H(k, q+2\pi)$, there is no such relation involving the momentum k parallel to the edge. Thus the Brillouin zone (BZ) is an infinite cylinder. Noting that $H(k,q)$ becomes trivial at large $|k|$, we can expect a vanishing Berry curvature there, so we may still define a Chern number for this BZ. The Hamiltonian $H(k,q)$ has 8 bands and is particle-hole symmetric – we compute the sum of the Chern numbers of the 4 negative-energy bands, for which we use the non-Abelian version of the algorithm from (6). We use the distribution in Eq. 7 of the main text to sample $\mathbf{f}^1, \mathbf{f}^2, \mathbf{g}^{1,2}$ and $\mathbf{g}^{2,3}$, while we choose $\mathbf{v}^1 = \mathbf{v}^2 = \mathbb{1}_4$ throughout as we observe no qualitative change in the distribution when speeds are allowed to vary.

A. Adding top modes. The Chern number argument above considers only a subset of the modes at the interface. The others must be added by hand, which we always do assuming a strong local coupling. Near the edge, there is the additional mode ψ_0 – when combined with the modes originating from the oscillating region via H_{c0}, we find that there are $\mathcal{C} - 1$ upstream Majorana modes near the edge. Further in the bulk, we find $\mathcal{C}$ downstream modes if N is even and $4 - \mathcal{C}$ upstream modes if N is odd. In the latter case, the N-th quadruplet interacts with the $\mathcal{C}$ chiral modes via $\mathbf{g}^{N-1,N}$. Note that in all cases, the total central charge is independent of $\mathcal{C}$ and matches the value mandated by the physical edge.

To match experimental results without fine-tuning, we require that c_{Edge}, the central charge of all the modes near the edge (including the charged boson, but not including the deep modes) obeys $|c_{\text{Edge}}| = \frac{1}{2}$ for interfaces with both $\nu = 2$ and $\nu = 3$ IQHE. It may be checked that this only holds for the $\mathcal{C} = 2$ state. In Fig. S5 we take a representative $\mathcal{C} = 2$ state with N even and add the top chiral Majorana mode. As the interaction strength H_{c0} is increased, we see how the top mode gaps with the two edge modes of H_{4N}. Crucially, this has no effect on the two chiral modes deeper in the bulk. Finally, the single remaining Majorana mode near the edge must have momentum $k = 0$ while the two deeper modes have $k = \pm k_0$ – this momentum mismatch is part of the reason why these modes struggle to equilibrate.

5. Transfer-matrix Analysis for Quenched Disorder

The above discussion has found that the Majorana mode system may likely be in a phase with $\mathcal{C} = 2$, which, when combined with the additional Majorana mode on the FQHE-vacuum interface results in an interface compatible with experimental observations. But this has relied on two assumptions – that all oscillations are the same (i.e. translational invariance perpendicular to the edge) and that the system is translationally invariant parallel to the edge. Although the Majorana interactions we discussed are not solely due to quenched disorder, its inclusion could significantly affect the outcome of our calculations. The topological nature of the result, however, suggests some degree of stability against perturbations. In this section, we simulate systems where both forms of translational invariance are explicitly broken by quenched randomness. To do this, we take a finite system and use a transfer-matrix method analogous to the methods used in (7). For a system of M Majorana modes $\psi_1, \ldots, \psi_M$, the Lagrangian in real space may be written as (summation understood)

$$\mathcal{L} = i\psi_a \left(\partial_t + v_a \delta_{ab} \partial_x - M_{ab}(x)\right) \psi_b \quad [7]$$

Here, v_a are the signed velocities of the modes, while $M_{ab}(x)$ is the real skew-symmetric interaction matrix which is now allowed to depend on position x. Instead of a smooth variation of $M(x)$, we discretize the space into intervals separated by points $\{x_n\}$. For $x_{n-1} < x \leq x_n$, take $M(x) = M_n$. Then, we have the full system Lagrangian

$$L = \int \mathrm{d}x \mathcal{L}(x) = \sum_n \int_{x_{n-1}}^{x_n} \mathrm{d}x \Psi^T \left(i\partial_t + iV\partial_x - iM_n\right) \Psi \quad [8]$$

where $\Psi = (\psi_1, \ldots, \psi_M)$ and $V_{ab} = \delta_{ab} v_a$ is the diagonal matrix of the signed velocities. We are interested in the low-energy physics, so we may set $\partial_t \Psi = 0$ in the Lagrangian. Then the mean field is simply read off $\partial_x \Psi = (V^{-1}) M_n \Psi$. We

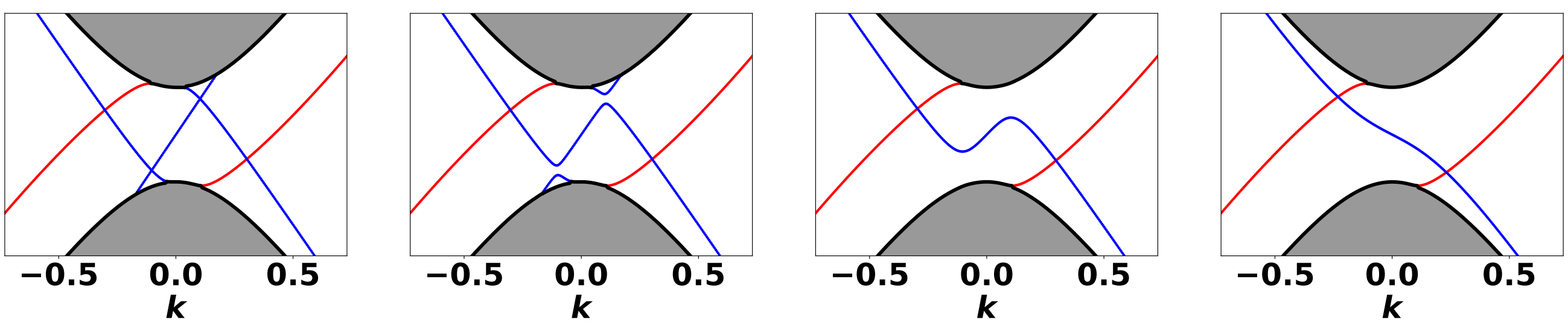

Fig. S5. Adding the top mode to an even-N state with $\mathcal{C} = 2$ to model a Pf-2 (aPf-3) interface. Blue modes are at the system edge while red modes are deeper in the bulk. In the leftmost frame, the top mode (positive-sloped blue line) is not interacting with the rest. From left to right, the interaction strength H_{c0} of the top mode with the rest is increased and the modes gap to produce one upstream edge Majorana mode. Without edge reconstruction, we would only see one downstream mode at the system edge (one positive-sloping blue line). The Pf-3 (aPf-2) interfaces have the deep (red) mode directions reversed.

assume all velocities to be nonzero, so V is invertible. The evolution along the x-axis is in general not unitary if there are counter-propagating modes – we may solve for the evolution to give

$$\begin{aligned}\Psi(x_K) = \exp\left(\left[x_K - x_{K-1}\right] V^{-1} M_K\right) \exp\left(\left[x_{K-1} - x_{K-2}\right] V^{-1} M_{K-1}\right) \ldots \\ \ldots \exp\left(\left[x_1 - x_0\right] V^{-1} M_1\right) \Psi(x_0) = T_K \Psi(x_0).\end{aligned} \quad [9]$$

This defines a transfer matrix T_K. We are physically interested in the limit of $K \to \infty$, where we keep the difference of consecutive x_n at order a few ℓ_B, or shorter. A mode localized over a lengthscale ξ shows up as an eigenvalue of magnitude $|\lambda| \sim e^{\pm(x_K - x_0)/\xi}$ in T_K. So the eigenvalue associated with a localized state may be either exponentially large or exponentially small in the distance over which the transfer matrix is computed. Delocalized modes should have eigenvalues with $|\lambda| = 1$.

To make the large-K limit numerically accessible, we use the Lyapunov-exponent-based algorithm of (7). Let $T_n = \exp\left(\left[x_n - x_{n-1}\right] V^{-1} M_n\right)$ be the transfer matrix associated with the n-th interval. If the elements of T_n are not too large, we can multiply multiple consecutive transfer matrices to get $\mathcal{T}_{(n+r,n)} = T_{n+r-1} T_{n+r-2} \ldots T_n$. The number r of matrices multiplied should be as large as possible while keeping a specified numerical precision. The main idea is to use $\mathcal{T}_{(n+r,n)}$ to act on a set of eigenvector guesses, noting the growth rates λ_k (exponents $\epsilon_k = \log|\lambda_k|$). After that, Gram–Schmidt orthonormalization is performed and the process repeated until we see convergence of the Lyapunov exponents. An important quantity is the localization length of a mode, defined by $\ell_k = r\Delta x / \langle \epsilon_k \rangle$, where we assumed that the intervals are evenly spaced, $x_n - x_{n-1} = \Delta x \; \forall n$.

We take the matrix structure as in Eq. 6 and take the elements, which are now functions of position in a Gaussian distribution analogous to Eq. 7. But we allow for a finite correlation length between the matrix elements with an exponential decay,

$$\begin{aligned}\left\langle g^{\alpha_1,\alpha_1+1}_{l_1 l_2}(x) g^{\alpha_2,\alpha_2+1}_{l_3 l_4}(x') \right\rangle = \delta_{l_1 l_3} \delta_{l_2 l_4} R^{|l_1 - l_2 + s - 3|} e^{-|x-x'|/\xi_1} e^{-|\alpha_1 - \alpha_2|/\xi_2} \\ \left\langle f^{\alpha_1}_{l_1 l_2}(x) f^{\alpha_2}_{l_3 l_4}(x') \right\rangle = \delta_{l_1 l_3} \delta_{l_2 l_4} R^{|l_1 - l_2| - 1} e^{-|x-x'|/\xi_1} e^{-|\alpha_1 - \alpha_2|/\xi_2} \quad (l_1 < l_2,\; l_3 < l_4)\end{aligned} \quad [10]$$

where $\alpha_1 - \alpha_2 = 2n$ for $n \in \mathbb{Z}$. If $\alpha_1 - \alpha_2$ is odd, there is no correlation. Setting $\xi_1, \xi_2 \to \infty$ would recover the method of using topological invariants. The choice of an exponential decay of correlations in position space is one of many possibilities, which is chosen for its numerical convenience. Furthermore, we choose all the velocities to be of the same magnitude – we have observed that allowing them to vary has no significant effect on the results. Setting their magnitude to unity implies a choice of the unit of length $\sim \ell_B$.

We choose to focus on a finite system with 13 (14) Majorana mode quadruplets in order to model the aPf-2 (Pf-2) interfaces. We choose a correlation length of disorder $\xi_1 = 20$ and we assume $\xi_2 \sim 3$ – this is motivated by expecting ξ_1 and ξ_2 to be at the same scale (as the period of the oscillations is $\sim 6\ell_B$).

With this, we run the above simulation over a distance of $5 \cdot 10^5\ \ell_B$ to find the eigenvectors and localization lengths of various modes. When $s = 0$ and $R < 0.6$ or when $s = 1$ and $R < 0.4$, a particular structure emerges in the results – for both types of interface, three modes seem to be delocalized, while the rest are well-localized. Fig. S6 shows the example of $R = 0.2$ and $s = 1$ for both interface types – the eigenvector weights show a picture where there is some weight close to the edge and approximately double the weight near the bulk side. The localization lengths and their relative uncertainties are shown in the insets – for three modes in each case, the uncertainties are larger than unity, indicating a high likelihood that these modes are truly delocalized. These modes strongly stand out from the rest. Similar results are found for the values of s and R described above, as well as for different correlation lengths.

The eigenvectors found here are a linear combination of the three modes discussed – but note that on short length scales, they are degenerate under the application of T_K. In fact the Lyapunov exponent is zero to within uncertainty for all three modes within our calculation. As a consequence, we may take our modes as any desired linear combination of the three found here and in particular we may choose a basis where one mode is localized at the system and two are "deep" towards the bulk. We are only forced to consider the combinations found in Fig. S6 on length scales comparable to l_{eq} for these modes. This indicates that the modes mix over distances similar to l_{eq} which are $\gtrsim 10^4 \ell_B \sim 1000\ \mu$m, which is significantly longer than the lengths in experiments (tens of μm). Because the Lyapunov exponent is zero to within uncertainty, this is only a lower bound on l_{eq} and the actual equilibration length may be much larger. Other parameter ranges, such as $s = 2$ or having $R \sim 1$ do not show such results. But it should be noted that this assumes the parameters are entirely spatially random and of zero mean – in reality, they may have a large constant component with random noise superimposed. There is no symmetry prohibiting a non-zero $\langle \mathbf{g}^{\alpha,\alpha+1}(x)$ and $\langle \mathbf{f}^{\alpha}(x) \rangle$ on top of the zero-mean random values described by Eq. 10, meaning that one should generally allow for a non-zero mean. In such a case, the system may even more reliably display this behavior, provided that the constant component puts it into the $\mathcal{C} = 2$ phase prior to adding the random component. Standard results about the stability of topological invariants apply to a weakly-disordered case with a large $\langle \mathbf{g}^{\alpha,\alpha+1} \rangle$ and $\langle \mathbf{f}^{\alpha} \rangle$. But Fig. S6 shows that even a fully random, strongly disordered system can generically display the type of behavior discussed in the main text – an upstream mode near the system edge and two Majorana modes moved deeper towards the bulk. Because we presently do not have a direct mapping between the microscopic Hamiltonian and the parameters $\mathbf{g}, \mathbf{f}$, it is difficult to establish whether the mean value $\langle \mathbf{g}^{\alpha,\alpha+1}(x) \rangle$, $\langle \mathbf{f}^{\alpha}(x) \rangle$ or the variance (Eq. 10) dominates. But our results show that the picture of a PH-Pf strip near the edge can be obtained robustly in either case without requiring any fine-tuning.

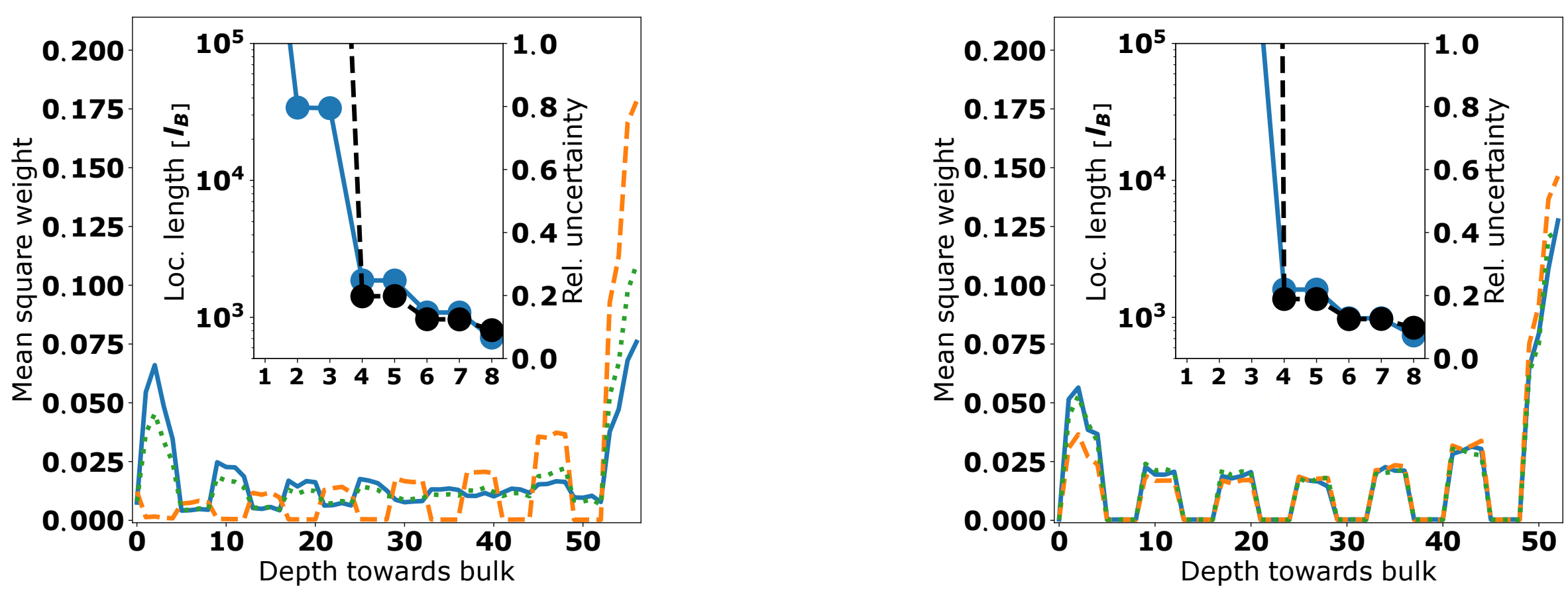

Fig. S6. The square weights of the three delocalized weights on both interface types. The left plot is the Pf-2 interface with 14 quadruplets of Majorana modes, while the right plot is the aPf-2 interface with 13 quadruplets of Majorana modes. Insets show localization lengths (solid blue) and their relative uncertainties (dashed black) for the different modes. In both cases, the first three modes have a very large relative uncertainty and with a localization length larger than others by an order of magnitude. The weights of these three eigenmodes of very large localization length are shown on the main axes.

A. Random operators and their scaling properties. Say that the Lagrangian includes a term $\xi(x)\mathcal{O}(x,t)$ where $\mathcal{O}$ is an operator of scaling dimension Δ and $\xi(x)$ is spatially random noise with ensemble average $\langle\xi(x)\xi(x')\rangle_{\text{dis}} = W\delta(x-x')$. The scaling dimension Δ is defined by requiring $\langle\mathcal{O}(x,0)\mathcal{O}x,t\rangle \sim t^{-2\Delta}$ for t large. Then, to the lowest order, the RG-flow equation is (8–10)

$$\frac{\mathrm{d}W}{\mathrm{d}l} = (3-2\Delta)\,W, \qquad [11]$$

showing that operators with $\Delta \leq \frac{3}{2}$ are relevant. Such spatially random operators are used to include disorder in our calculations and they are generally what leads to thermal equilibration of the modes. We may follow the reasoning of (8), where Eq. 11 is used to show that an operator with scaling dimension Δ and a random prefactor will lead to equilibration of the edge modes over a distance $l_{\text{eq}} \sim l_c\,(l_T/l_c)^{2\Delta-2}$ – since l_T diverges at low temperatures, operators with $\Delta > 1$ will equilibrate weakly as the temperature is lowered. For low enough temperature, l_{eq} surpasses the experimental system size and then the modes fail to equilibrate. This growth in l_{eq} and consecutive transition into non-equilibrium is more pronounced when Δ is larger. For non-random operators the scaling is different, $l_{\text{eq}} \sim T^{3-2\Delta}$. This is generally shorter than for the same operator with a random prefactor, so we might expect it to dominate. But a point to consider is that nonrandom operators have to conserve energy and momentum, which is not possible for an operator connecting two modes with different velocities (8). This significantly suppresses the contribution of nonrandom operators to equilibration. Of course, a microscopic value of W must still be input to the theory. Assuming that independent scatterers are a distance l_{Disorder} apart and that each has dimensionless amplitude S (the pre-factor in front of the scattering term beyond the necessary dimensionful factors, expected to be $\mathcal{O}(1)$), it can be shown that

$$l_{\text{eq, random}} \sim l_{\text{Disorder}}\frac{1}{S^2}\left(\frac{l_T}{l_c}\right)^{2\Delta-2}, \qquad [12]$$

with $l_T = \frac{v}{T}$ the thermal length. This form proves to be the most convenient in what follows.

6. Approximate experimental parameters

While the different experiments (11–14) differ in size and exact details, all generally have contacts of size $\sim 10\ \mu$m, which is also roughly the scale of separation between the contacts over which the edge modes propagate. The experiments are done around $B \sim 5$ T which puts the magnetic length at $\ell_B \sim 12$ nm. The experiments are done around $T \sim 15$ mK. While the exact velocities of the edge modes are not known, taking the Majorana mode velocity $v_\psi \sim 10^6$ cm/s and charged mode velocity $v_\phi \sim 10^7$ cm/s seems reasonable (10, 13, 15, 16). This puts the thermal length at $l_T = \frac{\hbar v_\psi}{k_B T} \sim 5\ \mu$m for the Majorana modes, and at $\sim 50\ \mu$m for the charged modes. The length scale associated with pairing in the states at $\nu = \frac{5}{2}$ is $l_c \sim 8\ell_B \sim 100$ nm (17). There is always some disorder present as the semiconductor (for example GaAs) in which the experiment is performed must be doped to be able to put electrons in the 2D quantum well in which the FQHE state lives. From the depth profile shown in (12), we may estimate that most of the disorder is situated ~ 100 nm above/below the state so we may take this to be the disorder correlation length (17). Doping densities range around 10^{11} cm^{-2}, which corresponds to a mean spacing ~ 30 nm between dopants.

7. Decoupling of the inner Majorana modes

Consider two Majorana modes, where tunneling between them is allowed. When the operator $\mathcal{O}$ is taken as Majorana mode tunneling $\mathcal{O} = S\psi_a\psi_b$, we can see that $l_{\text{eq}} \sim l_{\text{Disorder}}/S^2$ due to the scaling dimension $\Delta_{\mathcal{O}} = 1$. When propagating over lengths much shorter than l_{eq}, the modes are effectively thermally decoupled. They are thermally coupled over lengths larger than this scale. While a short-ranged impurity may be represented by a term $S\Gamma l_c\delta(x)\mathcal{O}(x)$ in the Lagrangian (here S is taken as dimensionless and Γl_c is the factor required on dimensional grounds), it turns out to be beneficial to also consider impurities extended over a distance w_D which may be represented by a term $\Gamma f(x)\mathcal{O}(x)$ where $f(x=0) \sim 1$ and $f(x) \to 0$ as $|x|/w_D \gtrsim 1$. It turns out that the argument above continues to hold provided that $w_D \ll l_T$, where we simply have to make the replacement

$$S \to \left|\frac{1}{l_c}\int \mathrm{d}x f(x)\right|. \qquad [13]$$

As discussed above, experiments generally have $l_T \sim 5\ \mu$m, while disorder is only correlated on scales ~ 100 nm.

With these pre-requisites, we are ready to tackle the problem of the three Majorana modes which survive in our system after the rest have been gapped out by the inter-wire couplings. We will assume that the system is in the $\mathcal{C} = 2$ phase. In that case, the edge is effectively described by a downstream $\nu = \frac{1}{2}$ chiral boson ϕ, an upstream Majorana mode χ localised at the edge and two inner Majorana modes $\psi_{1,2}$ propagating upstream (downstream) at aPf-2/Pf-3 (aPf-3/Pf-2). These may be combined into the Lagrangian

$$\mathcal{L} = \frac{2}{4\pi}\partial_x\phi(-i\partial_\tau + v_\phi\partial_x)\phi + \chi(\partial_\tau - iv_\chi\partial_x)\chi + \Psi^T(\partial_\tau - isv_\psi\partial_x + v_\psi k_0\sigma_y)\Psi. \qquad [14]$$

Where $s = 1$ ($s = -1$) for aPf-2 (Pf-2) and $\Psi = (\psi_1, \psi_2)^T$ is a vector containing the two inner Majorana modes. Note the term $\Psi^T v_\Psi k_0\sigma_y\Psi = -2ik_0\psi_1\psi_2$ – as discussed, this gives a finite momentum $k = \pm k_0 \sim \ell_B^{-1}$ to the modes.

To show that this edge model can explain the observed edge thermal conductance, we first argue that due to a combination of the lack of wavefunction overlap and momentum mismatch the inner modes entirely decouple from the thermal transport in

experiment – not only do they not equilibrate with the other modes, but they do not equilibrate with the thermal contacts either.

The most relevant operator that could lead to equilibration between the inner modes and the edge is Majorana mode tunnelling $\mathcal{O} = \chi\psi_{1,2}$. It has scaling dimension $\Delta = 1$, which according to Eq. 11 makes it RG-relevant. Consider the general tunnelling perturbation

$$\mathcal{L}_{\text{tunnel}} = i\frac{\sqrt{v_\psi v_\chi}}{l_c} G\chi F^T(x)\Psi \quad [15]$$

to Eq. 14. Here, $F(x) = (f_1(x), f_2(x))^T$ represents the coupling of both the inner modes to the edge mode with $f_{1,2}$ smooth functions varying over a distance $\sim w_D$. Here, G indicates the reduction in the tunnelling amplitude between the two modes due to the lack of wavefunction overlap – this is the factor that depends on the number of quadruplets, N. Given that we know the edge modes are localized, we may expect $G \sim e^{-N/\xi}$ for some localization length ξ, but this implicitly assumes disorder only allows nearby modes to interact – there might be some longer-range disorder, leading to a less sharp reduction in G as a function of N. We must first remove the non-random coupling between $\psi_{1,2}$ in Eq. 14 by an appropriate wavefunction transformation. Letting $\Psi = R(x)\tilde{\Psi}$ with $R(x) \in SO(2)$, such that $is\partial_x R(x) = k_0\sigma_y R(x)$, so that $R(x) = \exp(is\sigma_y k_0 x) \in SO(2)$, the final term in Eq. 15 becomes $\tilde{\Psi}\left(\partial_\tau - isv_\psi\partial_x\right)\tilde{\Psi}$, so the interaction term has been removed. In Eq. 15, $F(x)$ is replaced by

$$\tilde{F}(x) = \begin{pmatrix}\tilde{f}_1(x)\\ \tilde{f}_2(x)\end{pmatrix} = \begin{pmatrix}\cos(k_0x)f_1(x) - \sin(k_0x)sf_2(x)\\ \cos(k_0x)f_2(x) + \sin(k_0x)sf_1(x)\end{pmatrix}. \quad [16]$$

These transformed forms of the functions $\tilde{f}_{1,2}$ can then be used in Eq. 13. While exact details depend on the form chosen for $f_{1,2}$, we expect the general conclusions to hold for all slowly varying functions f peaked around $x = 0$ and with width w_D. We choose to parametrize the disorder by a Lorentzian, letting $f_1(x) = f_2(x) = S\frac{w_D^2}{x^2+w_D^2}$. This gives

$$l_{\text{Edge-inner}} \sim \frac{1}{G^2S^2} l_{\text{Disorder}} \frac{l_c^2}{w_D^2} e^{2w_Dk_0}. \quad [17]$$

It is evident that if w_Dk_0 is large, the correlation length becomes too big to equilibrate in practice. A Gaussian $f_{1,2}$ would lead to $\sim e^{(w_Dk_0)^2}$ which has even more suppression. While the value of k_0 is not known, we expect it to be $k_0 \sim \ell_B^{-1}$. So equilibration due to disorder with large w_D is exponentially suppressed in w_D/ℓ_B and will thus be ineffective. If $k_0 = 0.7\ell_B^{-1}$ and if we take $l_{\text{Disorder}} = w_D = 100$ nm $\sim 8\ell_B$ for these lengths and if we take $S = 1$ and $G = 1$ (the second being significantly too large), we still see $l_{\text{Edge-inner}} \sim 3 \cdot 10^3$ μm, which is over 10^2 times larger than typical experiments. But this has not yet taken into account the suppression due to the lack of wavefunction overlap – in the case where disorder introduces long-range interactions, G is not necessarily exponentially small in the number of quadruplets N, but it may obey some power law – for example the Coulomb-like $G \sim 1/N$. Consider this longer-range power law first. In the Coulomb case, another factor of $N^2 \gtrsim 10^2$ is introduced into the equilibration length, pushing it to 10^4 times larger than typical experiments – now even if the impurity strength S is larger, it is unlikely that such disorder can equilibrate the system. In fact we may estimate the strength of S as follows: the disorder is due to charged donors which are $h_{\text{dis}} \sim 100$ nm away from the sample and the typical spacing between them is also ~ 50 nm (12). We may thus expect that each point feels the potential of roughly one donor. The presence of these donors then alters the local chemical potential via the Coulomb interaction by $\Delta\mu \sim \frac{e^2}{4\pi\epsilon\epsilon_0 h_{\text{dis}}} \sim 10\ v_\psi/l_c$, using $\epsilon \sim 13$ as is appropriate for GaAs (16). If the modes were charged, this would suggest that $S \sim 10$ should be taken in Eq. 17, but since the modes are uncharged, they may interact more weakly with these fluctuations in the background electrical potential, potentially justifying a smaller S. Even with $S \sim 10$ and the other parameters as above, Eq. 17 suggests $l_{\text{Edge-inner}} \sim 8 \cdot 10^2$ μm, which as we see later can still be long enough for the modes to not equilibrate in practice. For this Coulomb-based effect, using $f(x) = S\frac{w_D}{\sqrt{x^2+w_D^2}}$ might be more appropriate. This form leads to an equilibration length ~ 3 times larger than what is suggested by Eq. 17 for the Lorentzian. This leads us to believe that short-ranged disorder might be more effective at equilibration. In that case however we may expect G to be exponentially suppressed in N, ie $G \sim e^{-N/\xi}$ (ie proportional to the wavefunction overlap), again leading to a large equilibration length. Furthermore, disorder with correlation lengths below $w_D \sim 100$ nm is not expected anyway, since the layer of doping responsible for the disorder is vertically separated from the electron gas in experiments

This all shows we have good reason to believe the inner modes do not equilibrate well with the other edge modes – but such non-equilibration generally increases the thermal conductance of an edge (18). In order for the inner modes to fully decouple, they should also decouple from the floating contacts used to measure the conductance. We expect this to happen for reasons similar to the above. First, since the modes are deeper into the sample, they may not interact with the contacts as strongly. Second, we can again expect that the term coupling the contacts to the edge is smooth (since contacts are large compared to microscopic length scales). Taking the Fourier component at $\pm k_0$ will again reduce the amplitude. We now address the question of how we couple to the contact in more detail. We expect that the contacts couple via electron tunnelling. An electron operator at the FQHE edge is built from the Bose mode ϕ and a Majorana mode ψ, giving $\psi_{\text{el}} = \psi e^{2i\phi}$. Within our model, any interface contains three Majorana modes and any of these three could take the role of ψ, but as discussed above, the two deep Majorana modes have a vanishing wavefunction overlap with the edge Bosonic mode – this leads us to conclude that any process that includes tunnelling an electron into the charged mode and a deep Majorana should be significantly suppressed.

This leads us to assume that the two modes at the edge are coupled well to the contacts, while the two inner Majorana modes are not. The exact details of how much weaker the inner modes couple depend on the microscopic physics of the contacts. Crucially any term in the Lagrangian that couples to the contacts suffers from the same two bare suppression effects as the equilibration terms discussed above, which has led us to the estimate that the lengthscale associated with this coupling is at the same order of magnitude as the equilibration length between the deep modes and the edge.

A. Hydrodynamic edge simulations. We finally address the question of just how large the equilibration length needs to be in order to produce results consistent with experiment. We use a hydrodynamic model of the edge analogous to those used in (18, 19). The crucial change is that the models in Refs. (18) and (19) assume good equilibration of all modes with the contacts. We relax this constraint, simulating a periodic system as shown in Fig. S7(a), but allow for a finite equilibration length between each mode and the contacts. Motivated by the experiment, we simulate an edge of width $L = 15$ μm and contact width $W = 8$ μm in the setup shown in Fig. S7(a) (we use a two-terminal setup for simplicity, noting that the key physics of decoupling should continue to hold even for a four-terminal measurement). We assume the edge charged mode ϕ and edge Majorana χ both equilibrate well with the contacts and assign a length of equilibration l_{Edge} between ϕ and χ. For the inner Majorana modes, we assume a length of equilibration l_{inner} between them and the edge Majorana mode χ. We assume that there is a similar lengthscale associated with their coupling to the floating contacts – for concreteness, we take the equilibration length between the inner modes and the contacts to be exactly l_{inner}. Considering both the cases where the inner Majorana modes propagate downstream and upstream to simulate aPf-3 and aPf-2, we find the results in Figure S7(b). We see that for $l_{\text{inner}} \gtrsim 10^2$ μm and for $l_{\text{Edge}} \lesssim 5$ μm, the conductance matches the experiment to within uncertainty on both interfaces. Our calculations have shown that $l_{\text{inner}} \gtrsim 10^2$ μm is realistic for the physical system. What remains is estimating l_{Edge}, the equilibration length between the charged boson ϕ and the edge upstream Majorana mode χ. This problem is exactly the same as the question of equilibration of an isolated PH-Pfaffian interface, the detailed mechanism behind which remains open. As has been discussed in Ref. (8), the most RG-relevant operator equilibrating the two modes is $\mathcal{O}_{\text{PHPf}} = (\partial_x\phi)\chi\partial_x\chi$ with scaling dimension $\Delta = 3$. The high scaling dimension means that perturbative calculations such as Eq. 12 necessarily predict a large l_{Edge}, especially at low temperature where $l_T \gg l_c$. For the experimental parameters, a perturbatively weak term $\xi(x)\mathcal{O}_{\text{PHPf}}(x)$ is not enough to guarantee $l_{\text{Edge}} \lesssim 5$ μm. Setting dimensionless quantities to order unity in Eq. 12 and using the parameters in Sect. 6, we find $l_{\text{Edge},\ \mathcal{O}_{\text{PHPf}}} \sim 6 \cdot 10^5$ μm, significantly larger than any experimental scale. If the physical edge in the experiment is to equilibrate, the mechanism behind it must clearly be significantly stronger than this perturbative scaling calculation suggests. We defer a more detailed microscopic understanding of why an isolated PHPf interface seems to equilibrate more strongly than standard scaling arguments imply to future work. We note that this problem is not unique to our proposed picture – the same question must be addressed even in a PHPf bulk picture, since that scenario also only matches experiment if the two edge modes ϕ and χ are brought into thermal equilibrium.

This mechanism of the two inner modes decoupling means that the noise profile is also equivalent to the PH-Pf profile. This is in appealing accord with noise measurements that agree with a PH-Pf-like state (13, 14, 18). Furthermore, any experiment which does not take special care to couple these inner modes to the physical system edge will not be able to differentiate between a genuine PH-Pf state and the mechanism proposed here, which could have either Pf or aPf as the bulk state.

References

1. N Read, D Green, Paired states of fermions in two dimensions with breaking of parity and time-reversal symmetries and the fractional quantum Hall effect. *Phys. Rev. B* **61**, 10267–10297 (2000).
2. KK Ma, MR Peterson, V Scarola, K Yang, Fractional quantum Hall effect at the filling factor $\nu = 5/2$ in *Encyclopedia of Condensed Matter Physics.* (Elsevier), pp. 324–365 (2024).
3. C Nayak, SH Simon, A Stern, M Freedman, S Das Sarma, Non-Abelian anyons and topological quantum computation. *Rev. Mod. Phys.* **80**, 1083–1159 (2008).
4. MP Zaletel, RSK Mong, F Pollmann, Topological Characterization of Fractional Quantum Hall Ground States from Microscopic Hamiltonians. *Phys. Rev. Lett.* **110**, 236801 (2013).
5. A Assouline, et al., Energy Gap of the Even-Denominator Fractional Quantum Hall State in Bilayer Graphene. *Phys. Rev. Lett.* **132**, 046603 (2024).
6. T Fukui, Y Hatsugai, H Suzuki, Chern Numbers in Discretized Brillouin Zone: Efficient Method of Computing (Spin) Hall Conductances. *J. Phys. Soc. Jpn.* **74**, 1674–1677 (2005).
7. F Merz, JT Chalker, Two-dimensional random-bond Ising model, free fermions, and the network model. *Phys. Rev. B* **65**, 054425 (2002).
8. KKW Ma, DE Feldman, Thermal Equilibration on the Edges of Topological Liquids. *Phys. Rev. Lett.* **125**, 016801 (2020).
9. CL Kane, MPA Fisher, Impurity scattering and transport of fractional quantum Hall edge states. *Phys. Rev. B* **51**, 13449–13466 (1995).
10. KKW Ma, DE Feldman, Partial equilibration of integer and fractional edge channels in the thermal quantum Hall effect. *Phys. Rev. B* **99**, 085309 (2019).
11. M Banerjee, et al., Observation of half-integer thermal Hall conductance. *Nature* **559**, 205–210 (2018).
12. B Dutta, V Umansky, M Banerjee, M Heiblum, Isolated ballistic non-Abelian interface channel. *Science* **377**, 1198–1201 (2022).

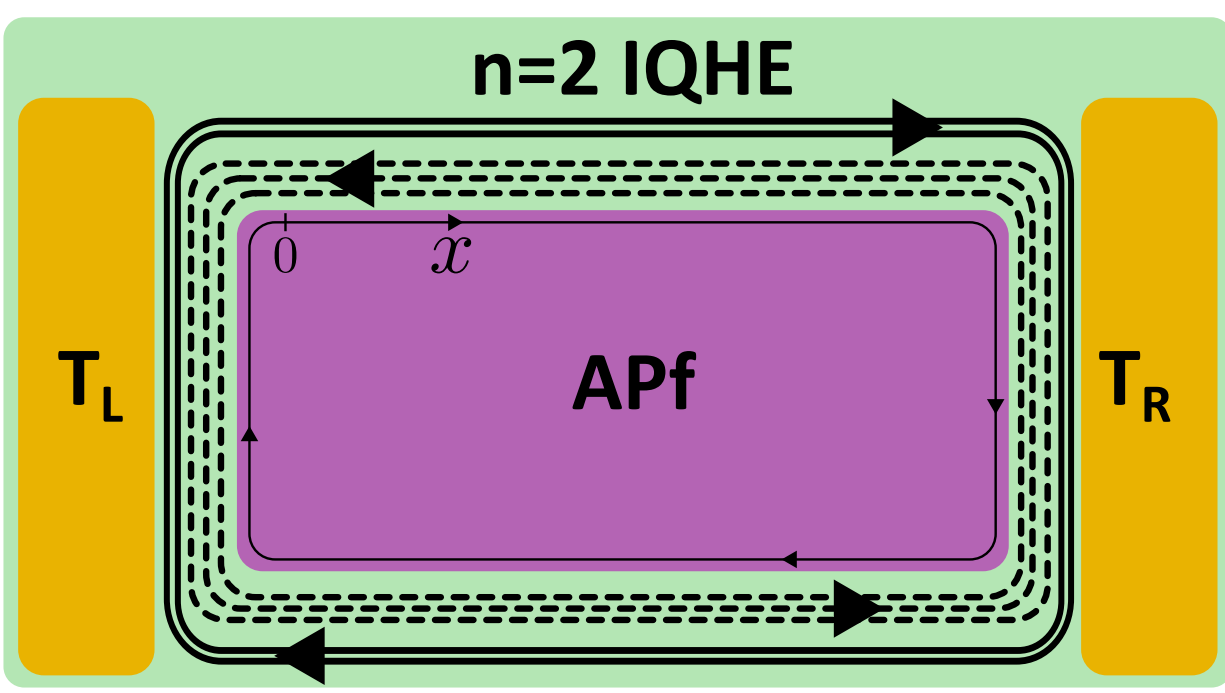

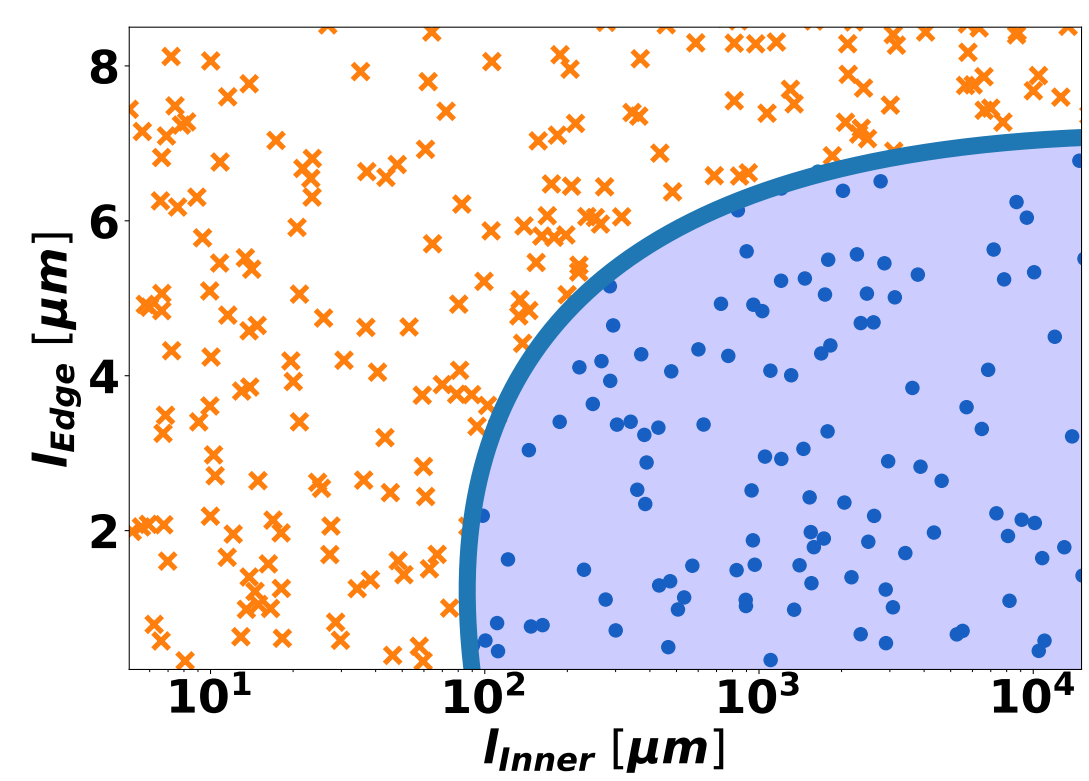

Fig. S7. (Left) The setup used in hydrodynamic simulation for the aPf-2 case. The modes encircle the aPf region and the coordinate x follows the modes in the downstream direction. Floating point contacts are at temperatures T_L and T_R. (Right) The range of equilibration lengths l_{Edge} and l_{inner} for which both the aPf-2 and aPf-3 interface have the experimentally observed value $\frac{G_Q}{\kappa_0 T} = 0.5 \pm 0.05$ is shaded. Not all simulated points are shown in favour of clarity.

13. AK Paul, P Tiwari, R Melcer, V Umansky, M Heiblum, Topological Thermal Hall Conductance of Even-Denominator Fractional States. *Phys. Rev. Lett.* **133**, 076601 (2024).
14. B Dutta, et al., Distinguishing between non-Abelian topological orders in a quantum Hall system. *Science* **375**, 193–197 (2022).
15. SH Simon, B Rosenow, Partial Equilibration of the Anti-Pfaffian Edge due to Majorana Disorder. *Phys. Rev. Lett.* **124**, 126801 (2020).
16. ZX Hu, EH Rezayi, X Wan, K Yang, Edge-mode velocities and thermal coherence of quantum Hall interferometers. *Phys. Rev. B* **80**, 235330 (2009).
17. W Zhu, DN Sheng, K Yang, Topological Interface between Pfaffian and Anti-Pfaffian Order in $\nu = 5/2$ Quantum Hall Effect. *Phys. Rev. Lett.* **125**, 146802 (2020).
18. M Hein, C Spånslätt, Thermal conductance and noise of Majorana modes along interfaced $\nu = \frac{5}{2}$ fractional quantum Hall states. *Phys. Rev. B* **107**, 245301 (2023).
19. C Nosiglia, J Park, B Rosenow, Y Gefen, Incoherent transport on the $\nu = 2/3$ quantum Hall edge. *Phys. Rev. B* **98**, 115408 (2018).
